# Giving superabsorbent polymers a second life as pressure-sensitive adhesives

**DOI:** 10.1038/s41467-021-24488-9

**Published:** 2021-07-26

**Authors:** P. Takunda Chazovachii, Madeline J. Somers, Michael T. Robo, Dimitris I. Collias, Martin I. James, E. Neil G. Marsh, Paul M. Zimmerman, Jose F. Alfaro, Anne J. McNeil

**Affiliations:** 1 Department of Chemistry, University of Michigan, Ann Arbor, MI USA; 2 School for Environment and Sustainability, University of Michigan, Ann Arbor, MI USA; 3 Materials Science Innovation—Corporate R&D, The Procter & Gamble Co., West Chester, OH USA; 4 Macromolecular Science and Engineering Program, University of Michigan, Ann Arbor, MI USA

**Keywords:** Polymer synthesis, Polymers

## Abstract

An estimated 6.3 billion metric tons of post-consumer polymer waste has been produced, with the majority (79%) in landfills or the environment. Recycling methods that utilize these waste polymers could attenuate their environmental impact. For many polymers, recycling via mechanical processes is not feasible and these materials are destined for landfills or incineration. One salient example is the superabsorbent material used in diapers and feminine hygiene products, which contain crosslinked sodium polyacrylates. Here we report an open-loop recycling method for these materials that involves (i) decrosslinking via hydrolysis, (ii) an optional chain-shortening via sonication, and (iii) functionalizing via Fischer esterification. The resulting materials exhibit low-to-medium storage and loss moduli, and as such, are applicable as general-purpose adhesives. A life cycle assessment demonstrates that the adhesives synthesized via this approach outcompete the same materials derived from petroleum feedstocks on nearly every metric, including carbon dioxide emissions and cumulative energy demand.

## Abstract (web summary)

For many polymers, recycling via mechanical processes is not feasible and these materials are destined for landfills or incineration. Here the authors show an open-loop recycling method for superabsorbent polymers that involves decrosslinking via hydrolysis, an optional chain-shortening step via sonication, and functionalizing via Fischer esterification.

## Introduction

Commercial polymers are ubiquitous, with a global annual production of approximately 368 million metric tons^1^, with about 75% of plastics disposed of after a single use^2^. Most of their feedstocks come from nonrenewable resources^3^. Although durability and strength are advantages of synthetic polymers, these properties are also responsible for their environmental persistence^4–6^. The synthetic polymer community created these impactful materials, and now, we must turn our attention towards technologies that facilitate their collection and recycling, to create a circular economy that is more sustainable^7^.

For decades, mechanical recycling has been used to recover some value from waste polymers, although recycling rates are low^2^ and the material quality is often reduced. Moreover, mechanical recycling cannot be used with polymers that do not reversibly melt (e.g., crosslinked polymers). An alternative recycling route known as chemical recycling was developed to address these challenges^8–11^. In closed-loop chemical recycling, chemical transformations are used to cleave the polymer into its monomers (depolymerization), or to create a polymer with equivalent function^12^. When closed-loop processes are unavailable, an alternative, known as open-loop chemical recycling can be utilized. Here, chemical transformations are used to convert waste polymers into other value-added materials, delaying their entry into the waste stream.

Sodium polyacrylate-based superabsorbent materials used in disposable diapers and feminine hygiene products have been challenging to recycle through either open- or closed-loop processes. The global annual production of this class of materials is estimated to be over 2 million metric tons, with disposable diapers claiming 74% of the market^13^. Unfortunately, most used diapers sit in landfills without substantial biodegradation, or are incinerated. To date, most diaper recycling efforts^14^ have focused on the cellulosic components, which can be biodegraded, incinerated to generate steam, pyrolyzed, or fermented to generate bioethanol^15^. In contrast, few studies have examined recycling of the sodium polyacrylate superabsorbent polymers. Mechanical recycling is not feasible because the crosslinks prevent melting. Decrosslinking has been reported using ozonolysis^16^, though not for the purposes of mechanical recycling. Closed-loop chemical recycling via depolymerization to monomer is also not currently feasible for these superabsorbent materials because side-chain degradation outcompetes depolymerization. For example, upon heating, polyacrylic acid is known to undergo dehydration and decarboxylation in bulk^17^ and solution^18^, respectively. Recent efforts to sidestep this degradation using microwaves and added radical initiators led to oligomeric products with 50–60% decarboxylation^19^. While catalytic depolymerization methods^20^ can, in principle, proceed without side-chain degradation, they have not yet been demonstrated for commercial sources of polyacrylic acid. Due to these challenges, the focus has been on re-use of recovered materials, rather than recycling; for example, the recovered superabsorbent polymers have been explored for enhancing the water retention properties of soil^21,22^.

**Fig. 1:**
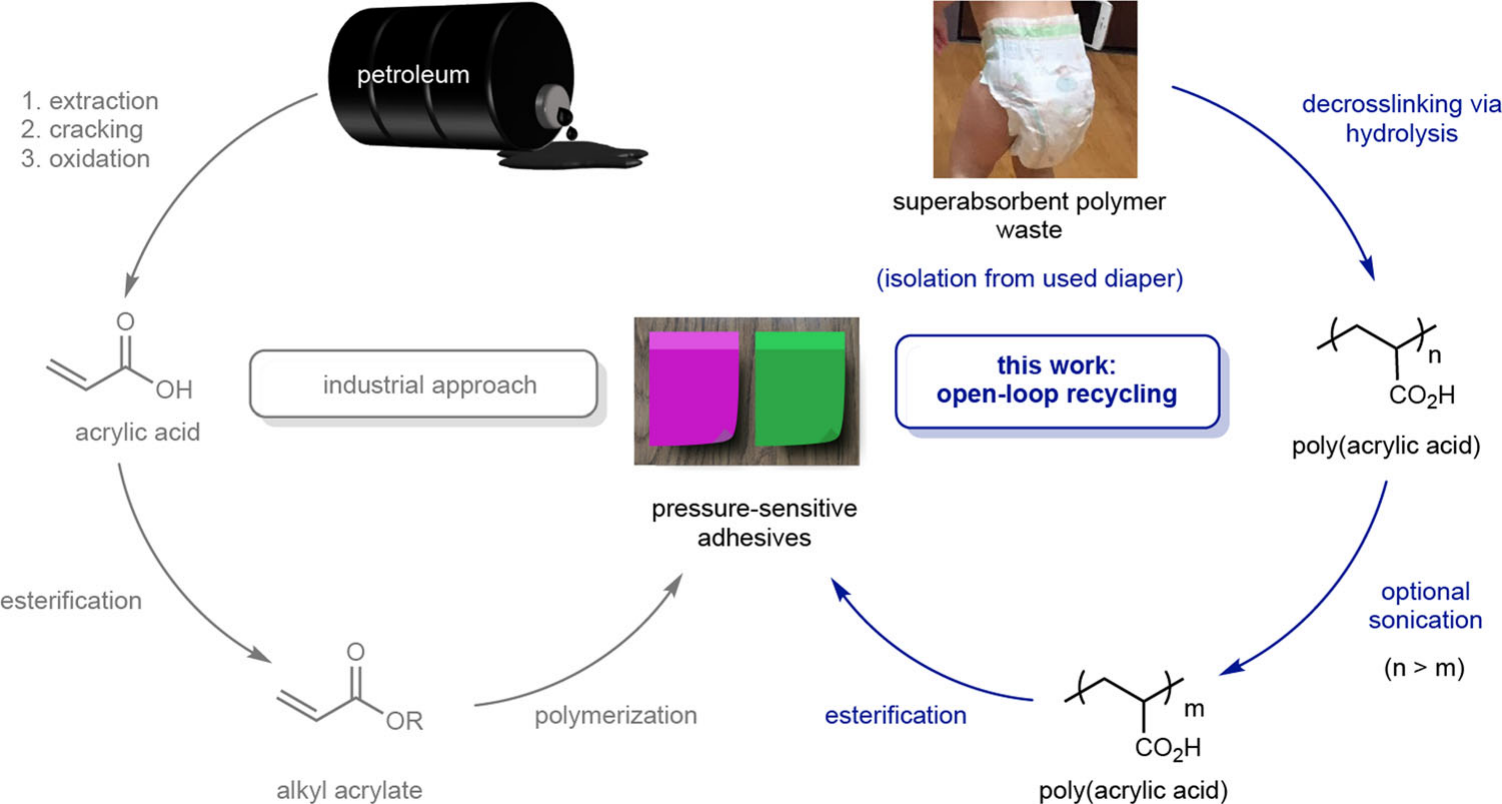
Synthetic routes to pressure-sensitive adhesives. Syntheses of pressure-sensitive adhesives from petroleum feedstocks (industrial approach) compared to waste diaper-based feedstock (open-loop recycling).

In this work, we report a mild and efficient synthetic route for open-loop recycling the acrylic-based superabsorbent material used in diapers. Specifically, using a 2 or 3 step synthetic process, the sodium polyacrylate is converted into a pressure-sensitive adhesive (PSA), which has a significant global market (expected to be $13 billion by 2023) (https://www.alliedmarketresearch.com/pressure-sensitive-adhesives-market). This approach was inspired by the similar structures of sodium polyacrylate (superabsorbent polymer) and the polyacrylates (pressure-sensitive adhesives) used in tapes, bandages, and sticky notes, among others^23^. Commercial polyacrylate-based PSAs are often accessed via petroleum-sourced monomers (Fig. 1)^24–26^. Instead, we envisioned a 2–3 step method from crosslinked sodium polyacrylate: (i) acid-catalyzed or base-mediated decrosslinking to generate linear polymers, (ii) optional sonication to lower the molar mass, and (iii) functionalizing via esterification to generate tack (Fig. 1). In any recycling effort, it is essential that a life cycle assessment^27^ is performed to determine the energy and environmental costs. We demonstrate herein that our open-loop recycling approach to pressure-sensitive adhesives from superabsorbent polymers outcompetes the petroleum-derived syntheses on nearly every metric, including global warming potential and cumulative energy demand. Moreover, the route involving just decrosslinking and esterification (i.e., no sonication) has the potential to be industrially scalable, providing a sustainable solution to a longstanding waste problem.

## Results and discussion

### Isolation from post-consumer waste

Globally, there have been significant efforts towards recycling the components of diapers^28^. For example, FaterSMART, a Proctor and Gamble (P&G) affiliated company, has developed and implemented a diaper recycling facility that includes used diaper acquisition, steam sterilization, shredding, and separation into the purified raw materials (cellulosics, superabsorbent polymer, and polyolefins)^29^. We have included these important steps in our life cycle assessments, however, our syntheses utilized the more readily accessible samples of superabsorbent polymer used to manufacture diapers at P&G.

### Decrosslinking via hydrolysis

**Fig. 2:**
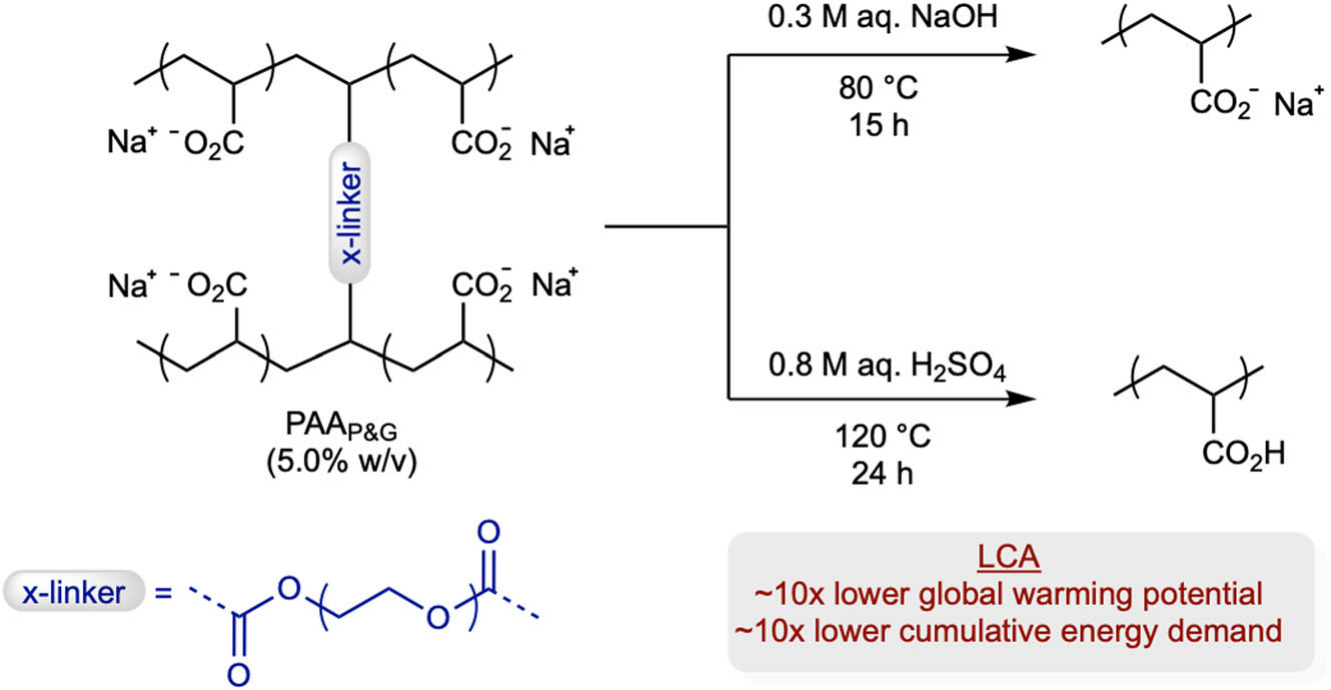
Decrosslinking via hydrolysis. Reaction conditions for base-mediated and acid-catalyzed decrosslinking reactions with LCA comparison.

The superabsorbent polymer provided by P&G is a sodium poly(acrylate) crosslinked via ~0.05 mol% poly(ethylene glycol) diacrylate co-monomer (PAA_P&G_)^30^. The crosslinks were first hydrolyzed using 0.3 M aq. NaOH and mild heating (Fig. 2). The initially heterogeneous reaction mixture becomes a homogeneous solution over time due to the chemical change from a superabsorbent gel-like substance into the soluble, linear polymer products. To monitor this process, we measured changes in the complex viscosity over time. Within the first 15 h, we observed a substantial drop (more than two orders of magnitude) in viscosity, after which point no further changes were observed. This result suggests that most crosslinks had been hydrolyzed within 15 h (Supplementary Fig. S1). For comparison, we also evaluated the hydrolysis using 0.8 M aq. H_2_SO_4_ with heating (Fig. 2). The complex viscosity again dropped several orders of magnitude over 24 h, at which point no further changes were observed, again suggesting that most crosslinks have been hydrolyzed (Supplementary Fig. S2). To determine whether the base-mediated or acid-catalyzed pathway was better, the two routes were compared using a cradle-to-product life cycle assessment (LCA, Supplementary Fig. S3). The acid-catalyzed hydrolysis outperformed the base-mediated hydrolysis by a factor of 10 on both global warming potential and cumulative energy demand. The resulting acidic polymer solutions were used directly in the subsequent experiments without any isolation steps.

### Chain-shortening via sonication

To access more than one type of pressure-sensitive adhesive, we needed a method to shorten the linear polymers obtained after the decrosslinking step. Sonication has previously been used to chain-shorten high-molar mass polymers with various backbone architectures^31,32^, including polyacrylic acid^33^, and has been used at scale^34^. Under ultrasound, solvodynamic shear forces cleave polymer chains into shorter fragments while maintaining the polymer’s chemical identity. The rate of chain scission during sonication is directly proportional to the amount of chain entanglement during sonication^35^. As a consequence, there is an intrinsic, limiting molar mass for each polymer below which further chain scissions are unlikely to occur. Experimentally, a plateau is observed in the plot of weight-average molar mass (*M*_w_) versus sonication time.

**Fig. 3:**
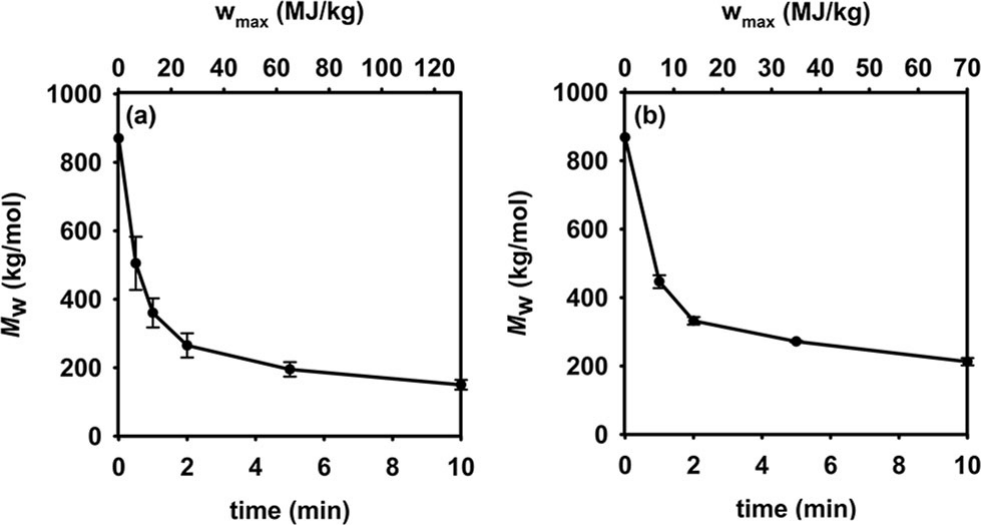
Changes in molar mass with sonication. Plots of weight-average molar mass (*M*_w_) versus time and maximum specific energy (*w*_max_) for sonicating PAA_P&G_ at **a** 2.5% w/v and **b** 5.0% w/v. Error bars are derived from an average of two experiments.

Sonicating 2.5% and 5.0% w/v solutions of decrosslinked PAA_P&G_ for 0–10 min using a 20 kHz sonication horn operating at full amplitude (100%) revealed rapid chain-shortening for the decrosslinked PAA_P&G_ (Fig. 3). At each time point, the maximum specific energy (*w*_max_) was calculated using the maximum power drawn from the outlet and the mass of added PAA (Supplementary Information pg S3), and the molar masses were determined using size-exclusion chromatography.

To achieve the necessary cohesive and holding strength for a PSA, the polymer (after esterification) should have a *M*_w_ > 400 kg/mol^25,36^. Considering this factor, the optimized conditions involved sonicating a 2.5% w/v solution for 1 min to give an *M*_w_ ~360 kg/mol, and a 5.0% w/v solution for 2 min to give a *M*_w_ of ~330 kg/mol^37^. The resulting chain-shortened PAA_P&G_ fragments were then dialyzed to remove excess acid, lyophilized, and then ground into a powder. The resulting polymers were isolated in ~97% yield (over the two steps). A life cycle assessment was used to compare the routes that involved (i) no sonication, (ii) sonication for 1 min (2.5% w/v) and (iii) sonication for 2 min (5.0% w/v), including the workup steps, and will be described in more detail below.

### Esterification of polymer fragments

Several routes for converting the polyacrylic acid into a polyacrylate were compared. Esterification of polyacrylic acid using alkyl halides under basic conditions had already been reported^38^. However, this process utilizes expensive solvents (dimethylsulfoxide or dimethylformamide) and stoichiometric quantities of base (tetramethylguanidine), both of which would likely be too costly for large-scale recycling of waste superabsorbent materials. In contrast, a common approach used in industry to convert acrylic acid into acrylate esters uses inexpensive alcohols as both the reagent and solvent^39,40^. However, this approach can lead to low yields due to competitive ester hydrolysis^41,42^ and catalyst deactivation by water^43^. To circumvent these challenges, the water by-product can be selectively removed via azeotropic distillation, or a large excess of alcohol can be employed^44,45^.

**Fig. 4:**
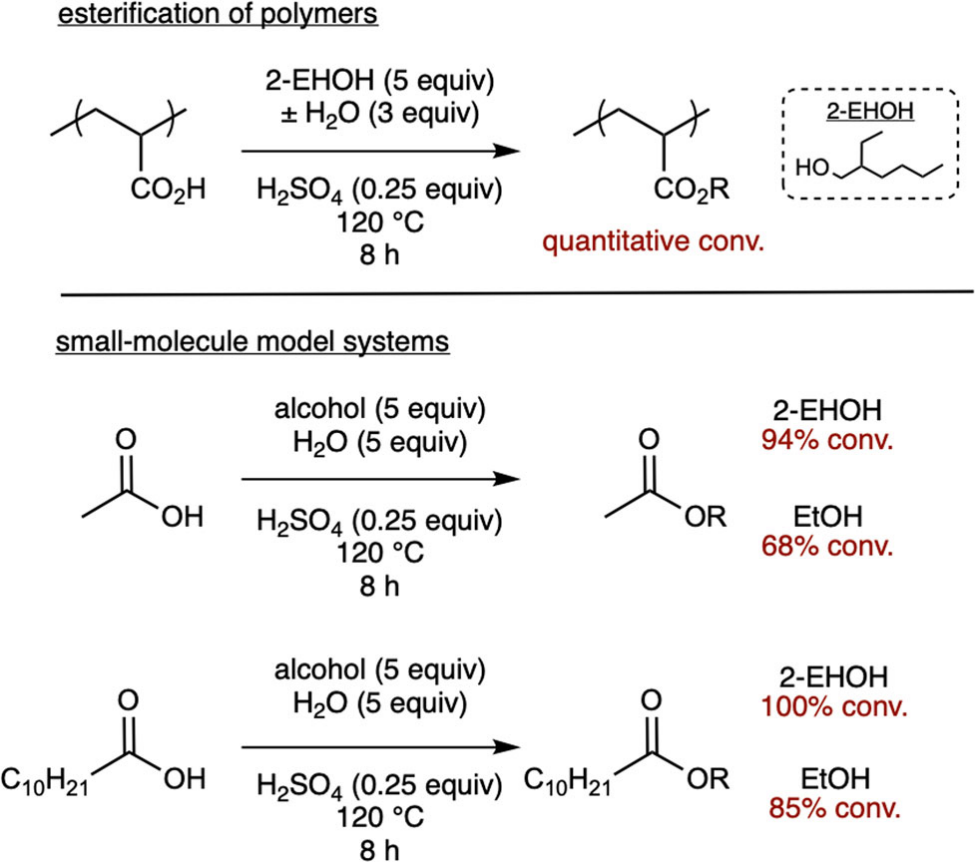
Acid-catalyzed esterification. Reaction conditions and yields for acid-catalyzed esterification of poly(acrylic acid), acetic acid, and undecanoic acid.

We hypothesized that we could potentially eliminate the need to remove water because both the polymer backbone and our alcohol solvent are hydrophobic. To interrogate this hypothesis, we measured the percent esterification under different conditions. For example, high degrees of esterification were observed via ^1^H NMR spectroscopy when using only 5 equiv. of 2-ethylhexanol and H_2_SO_4_ as a catalyst (Fig. 4). Surprisingly, even when excess H_2_O was intentionally added (3 equiv), high conversions were still observed (Supplementary Information pgs S15–S16). The results from both of these experiments demonstrate that the equilibrium lies far towards the esterification product.

To understand why esterification is so favored, we turned to small molecule model systems (Supplementary Information pgs S17–S26). To probe the role of both solvent and substrate hydrophobicity, we used two different substrates (acetic versus undecanoic acid) and solvents (ethanol versus 2-ethylhexanol). When acetic acid was reacted with 2-ethylhexanol/water, we observed a 94% conversion. In contrast, when acetic acid was reacted with ethanol/water, the conversion was only 68%. These results demonstrate that solvent hydrophobicity improves conversion to the ester. Next, undecanoic acid was esterified under the same conditions, yielding 100% ester for 2-ethylhexanol/water and 85% for ethanol/water. These results suggest that the substrate hydrophobicity also favors conversion to the ester. From these studies, we conclude that the quantitative esterification of the polymer results from the hydrophobic reaction environment created by the polymer backbone and the 2-ethylhexanol solvent.

To probe whether the hydrophobic side chains also push the equilibrium towards esterification, atomistic simulations were used (Supplementary Information pgs S27–S29)^46,47^. Briefly, nonamers of polyacrylic acid were used as a model system along with butanol Comparison of the reaction free energies was made between the first esterification and the final esterification. In both cases, the nonamers were solvated in a 3:1 butanol/water mixture to mimic the most challenging esterification conditions. The change in the Helmholtz free energy of esterification for these two steps (ΔΔA) was found to be −0.7 ± 0.1 kcal/mol. This value suggests that the increase in polymer hydrophobicity provides a significant thermodynamic driving force towards further esterification, perhaps counteracting the buildup of water that was otherwise expected to limit conversion.

### Characterizing the adhesive properties

**Fig. 5:**
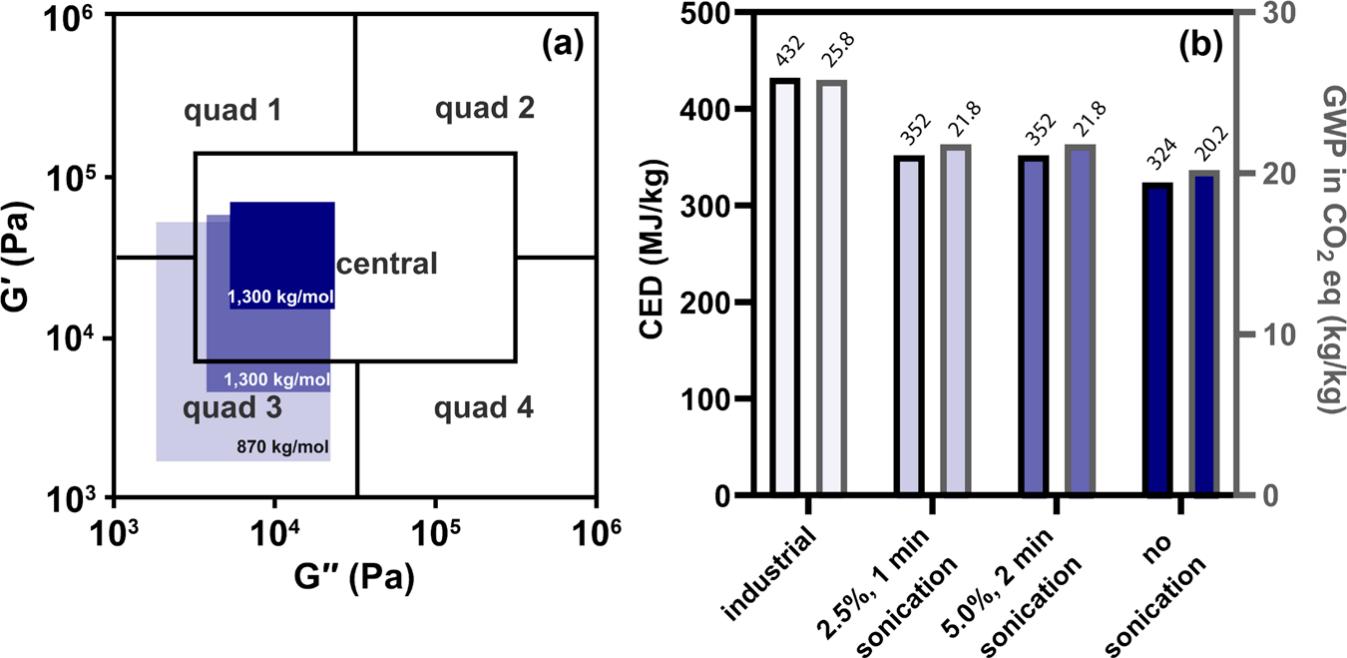
Adhesive properties and life cycle assessments. Plots of storage (G′) versus loss (G′′) moduli for poly(2-ethylhexylacrylate) (**a**), including visualization of Chang’s viscoelastic window, and of the cumulative energy demand (CED) and global warming potential (GWP) for each route (**b**).

The adhesive properties of the synthesized polyacrylates were evaluated using rheology and analyzed with respect to Chang’s viscoelastic window (VW), which classifies different adhesive types^48^. In this approach, the VW for each PSA is constructed from the dynamic storage (G′) and loss (G′′) moduli at representative bonding and debonding frequencies of 0.01 and 100 Hz, respectively. The corresponding VW for each adhesive is the rectangular region bounded by these four moduli. Chang noted that most existing PSAs appear between the G′ and G′′ bounds of 10^3^ and 10^6^ Pascals (Pa) at the aforementioned bounding frequencies, and can be grouped into the quadrants (and central region) highlighted in Fig. 5. The G′ at each frequency describes an adhesive’s resistance to shear, and this term generally increases in samples with more chain entanglements (e.g., with increasing *M*_w_). The G′′ at each frequency describes an adhesive’s ability to dissipate energy. Most consumer PSA-based products are found in either quadrant 3 or the central region (e.g., office tape, sticky notes, bandages, and removable labels), which is signified by low-to-medium G′ and G′′.

All the adhesives synthesized from PAA_P&G_ fall within quadrant 3 and the central region (Fig. 5)^49,50^. That is, the PSAs are soft enough to flow and wet a substrate at the bonding frequency, while hard enough to hold onto a substrate, and peel cleanly at the debonding frequency. As expected, the VWs are higher with larger *M*_w_, which is due to the increased chain entanglements. Overall, the viscoelastic properties of the synthesized PSAs suggest they would be useful for applications such as removable general-purpose adhesives, including tapes, bandages, and sticky notes. Excitingly, a viable central region pressure-sensitive adhesive is accessible without any sonication.

### Life cycle assessments

Life cycle assessments (LCA) were performed to assess the cumulative energetic and environmental impacts of producing 1 kg of adhesive via our open-loop recycling method. More specifically, we compared four different cradle-to-product LCA scenarios: poly(2-ethylhexylacrylate) production in the conventional industrial approach and three variations of the recycling process: (i) sonicating the 2.5% w/v polymer solution for 1 min, (ii) sonicating the 5.0% w/v polymer solution for 2 min, and (iii) no sonication of the 5% w/v polymer solution. All environmental data were gathered from experiments, literature, or the ecoinvent database (version 3.5)^51^, and implemented in SimaPro v. 9.0.0.48^50^, as described in detail within the Supporting Information (Supplementary Information pgs S36–S41). Several environmental impact categories were examined, but particular attention was paid to the global warming potential (GWP) and cumulative energy demand (CED). We found a 15.3% and 15.1% decrease in GWP when replacing the industrial route with either the 1 min or 2 min sonication scenario, respectively, and an impressive 21.5% decrease when sonication was avoided altogether. From an energetic standpoint, the CED is reduced by 18.8% and 18.6% with the sonication scenarios, and further reduced (by 24.8%) in the scenario without sonication. Combined, these data indicate that open-loop recycling of the superabsorbent poly(acrylic acid) to synthesize PSAs is both energetically and environmentally favorable compared to petroleum-derived syntheses in our assessment.

Given a growing emphasis on sustainability within the polymer industry, including calls to increase the circularity of polymer production^12^, this LCA provides an important metric for evaluating approaches to polymer recycling. At present, the environmental benefits of diaper recycling (including superabsorbent poly(acrylic acid) recovery) are dependent on the avoided material burdens^52^. One of the pitfalls of diaper recycling pilots is the low demand for recovered diaper materials, which depreciates the environmental potentiality of such endeavors^14^. Therefore, efforts to introduce synergy between superabsorbent poly(acrylic acid) recovery and PSA production may provide much-needed demand for diaper recycling end-products, which in-turn may improve environmental performance for both processes on a large scale.

To summarize, we developed a facile and potentially scalable method to synthesize commercially relevant PSAs by open-loop recycling poly(acrylic acid) sourced from a leading diaper manufacturer. The transformation relies on an (i) acid-catalyzed hydrolysis, (ii) optional chain-shortening via sonication, and (iii) a highly efficient esterification driven by hydrophobicity. Different PSAs were targeted simply by varying the sonication times. Moreover, the life cycle assessments, which utilized soiled diapers as the starting point, demonstrated that these open-loop recycling methods outperform the conventional routes from petroleum-derived feedstocks on nearly every metric, including for the key LCA metrics of global warming potential and cumulative energy demand. Because this recycling method should be amenable to waste polymer as the feedstock, it offers a more sustainable alternative to diaper disposal in a landfill or incineration.

## Supplementary information

Supplementary Information

## Data Availability

The materials, equipment, synthetic methods, as well as all processed data generated in this study are provided in the Supplementary Information file.
