# Supplementary material for: Giving superabsorbent polymers a second life as pressure-sensitive adhesives
Source: Nat Commun. 2021 Jul 26;12:4524. doi: 10.1038/s41467-021-24488-9 (PMC8313680; doi:10.1038/s41467-021-24488-9)
Supplement: Supplementary file 1 — Supplementary Information [file 41467_2021_24488_MOESM1_ESM.pdf]

Supporting Information for:

Giving Superabsorbent Polymers a Second Life as Pressure-Sensitive Adhesives.

P. Takunda Chazovachii,[1] Madeline J. Somers,[2] Michael T. Robo,[1] Dimitris I. Collias,[3] Martin I. James,[3] E. Neil G. Marsh,[1] Paul M. Zimmerman,[1] Jose F. Alfaro,[2] and Anne J. McNeil[1,4]*

[1]*Department of Chemistry, University of Michigan, 930 North University Avenue, Ann Arbor, Michigan, 48109, United States*
[2]*School for Environment and Sustainability, University of Michigan, 440 Church Street, Ann Arbor, Michigan, 48109-1055, United States*
[3]*Materials Science Innovation – Corporate R&D, The Procter & Gamble Co., 8611 Beckett Road, West Chester, Ohio, 45069, United States*
[4]*Macromolecular Science and Engineering Program, University of Michigan, 2800 Plymouth Rd, Ann Arbor, Michigan, 48109, United States*

Email: ajmcneil@umich.edu

Table of Contents

Supplementary Methods & Discussion S2

I. Materials S2

II. General experimental S3

III. Comparing base-mediated versus acid-catalyzed decrosslinking of $PAA_{P\&G}$. S5

IV. Evaluating polymer recovery and chemical structure after sonication S8

V. Monitoring chain-shortening and energy consumption at 2.5 and 5.0% w/v S9

VI. Fischer esterification studies S13

VII. Free-energy calculations S27

VIII. Esterifying $PAA_{P\&G}$ fragments to make PSAs S30

IX. Life cycle assessment S36

Supplementary References S42

Supplementary Methods & Discussion

I. Materials

All chemicals were used as received unless otherwise mentioned. Polyacyrlic acid (PAA) with molecular weight listed as 1,033 kg/mol (PAA_{SPP}) was purchased from Scientific Polymer Products. PAA_{SIGMA1} (listed as 240 kg/mol), PAA_{SIGMA2} (listed as 450 kg/mol), *p*-toluenesulfonic acid (*p*-TsOH), decanoic acid, undecanoic acid, 2-ethylhexanol (2-EHOH), sodium hydroxide (NaOH), sulfuric acid (H_2SO_4), and sodium nitrate were purchased from Millipore Sigma. Methanol (MeOH) was purchased from Fisher Scientific. Tetrahydrofuran (THF) was purchased from OmniSolv. Glacial acetic acid was purchased from Acros Organics. Deuterated solvents: chloroform ($CDCl_3$), pyridine-d_5, and deuterium oxide (D_2O) were purchased from Cambridge Isotopes. Sonicated polymer fragments were dialyzed in deionized (DI) water using Spectra/Por molecular porous membrane tubing (molecular weight cut-off: 3.5 kg/mol). Pressure vessels were purchased from Thomas Scientific. Jacketed beakers were purchased from Sigma Aldrich (cat#: Z202738-1EA).

Note that $PAA_{P\&G}$ is a commercial product, that was purchased from NIPPON SHOKUBAI Co. Ltd. and is called Aqualic CA™ L700. The degree of crosslinking is a trade secret, and not public information. However, according to a patent issued to NIPPON,[1] a representative absorbent resin contains 0.05 mol% polyethylene glycol diacrylate crosslinker relative to the acrylic acid monomer. In addition, the polymer is 75% neutralized (i.e., % sodium form) and contains ~0.5% moisture. Because there is only a small variability in crosslink density within commercial SAPs used in hygiene products, the reported decrosslinking conditions and reaction times should be similar (or even the same) for other SAP sources.

II. General experimental

Sonication – Sonication was performed at 100% amplitude (amp) using a Sonics and Materials Vibra-cell VCX 600 Ultrasonic Liquid Processor equipped with a 13 mm replaceable tip probe. A 3.5 cm inner diameter, 9 cm height jacketed beaker was used for all sonication procedures. Cold water (10–15 °C) was flowed through the jacket while stirring the polymer solution at 500 rpm. A thermocouple was immersed into the polymer solution to monitor temperature. The temperature was generally observed to increase from 10–15 °C to 45–50 °C during sonication. The power from the outlet was monitored using a kill-a-watt meter (#P4400). The maximum power (P_{max}) reading observed at the beginning of sonication was recorded. The maximum specific energy (w_{max}) for chain-shortening PAA of mass (m) for time (t) was determined using supplementary equation (1).

$$w_{\mathrm{max}}\ (\mathrm{J/kg}) = \frac{P_{\mathrm{max}}\ (\mathrm{W}) \times t\ (\mathrm{s})}{m\ (\mathrm{kg})} \qquad \text{Supplementary Equation (1)}$$

NMR Spectroscopy – Unless otherwise noted, ^{1}H and ^{13}C NMR spectra for all compounds were acquired at room temperature. Chemical shift data are reported in units of δ (ppm) relative to tetramethylsilane (TMS) and referenced with residual solvent. Multiplicities are reported as follows: singlet (s), doublet (d), doublet of doublets (dd), triplet (t), quartet (q), multiplet (m), and broad resonance (br). Residual water is denoted by an asterisk (*). For all ^{1}H NMR spectra of polymers, a 3.5 s acquisition time was used with a 10 s relaxation delay between each pulse.

Size Exclusion Chromatography (SEC) for $PAA_{P\&G}$ fragments – Sonicated PAA_{SPP} and $PAA_{P\&G}$ fragments were diluted (to 1–1.5 mg/mL) with 0.2 M $NaNO_3$ (aq)/ethylene glycol (99:1 v/v) and filtered through a Titan3™ Nylon syringe filter (0.45 μm) into a SEC vial.

Polymer molar mass (*M*) and dispersity (Đ) were determined by comparison with PEG/PEO EasiVial standards from Agilent at 40 °C in 0.1 M $NaNO_3$ (aq) on a Waters SEC (Waters 1515 Isocratic HPLC pump, 717plus autosampler, RI detector Model 214 and UV-PDA detector Model 487) equipped with a 250 Ultrahydrogel column (WAT011525) and a PL aquagel-OH MIXED-H column (PL1149-6800).

Dialyzing, free-drying, and grinding polymer fragments
After sonication, the polymer was dialyzed using DI water (~1 gallon), switching the DI water three times over 12–18 h. Then, the polymer was freeze-dried and ground to a fine powder using a mortar and pestle. More specifically, while wearing cryogenic gloves, a piece of freeze-dried polymer was put into a mortar, which was then immersed into a bath of liquid N_2. A small amount of liquid N_2 was poured into the mortar and the polymer was ground using a pestle. The fine powder was immediately transferred to a 20 mL vial and held under high vacuum for 10 min as the polymer warmed to rt to avoid water condensation.

SEC for polyacrylate-based PSAs (pressure-sensitive adhesives) – The synthesized PSAs were dissolved in THF (1 mg/mL) with mild heating and filtered through a PTFE filter (0.45 μm) into an SEC vial. Polymer molar mass (*M*) and dispersity (Đ) were determined at 40 °C in THF on a SEC: Malvern Viscotek GPCMax VE2001 equipped with two Viscotek LT-5000L 8 mm (ID) × 300 mm

(L) columns, and Viscotek TDA 305 and Viscotek RI detectors. Apparent molar masses were calculated using EasiVial PMMA standards (spanning 690–1,944,000 g/mol) provided by Polymer Laboratories.

Rheology – All rheological measurements were taken on an AR2000ex rheometer (TA Instruments). A 40 mm stainless steel parallel plate was used to run frequency sweeps for decrosslinked $PAA_{P\&G}$. First, an aliquot of the reaction mixture (1.2 mL) was added to the bottom plate. The upper plate/geometry was lowered to a gap of 605 μm. While the geometry rotation was locked, excess sample was wiped away using a custom-built glass piece that trims the excess sample along the circumference of the geometry. Then, the plate was lowered to the desired gap of 600 μm. For reference, see this TA instruments video (https://www.youtube.com/watch?v=kFiVLSzjUlc). DI water (1.2 mL) was added into the solvent cavity on the plate followed by the solvent trap. For reference, see this TA instruments video (https://www.youtube.com/watch?v=OQmAtdvYrws). The frequency sweeps were performed between 0.1 and 100 Hz at 1% strain and 25 °C. This process was repeated at least twice for each sample with cleaning and calibration between runs.

A 25 mm serrated parallel plate was used to run frequency sweeps for the PSAs. PSA (~600 mg) was loaded to achieve a 1,250 μm layer thickness. The frequency sweeps were performed between 0.01 and 100 Hz at 1% strain and 25 °C. This process was repeated at least twice for each sample with cleaning and calibration between runs.

III. Comparing base-mediated versus acid-catalyzed decrosslinking of $PAA_{P\&G}$.

Monitoring decrosslinking at 0.3 M NaOH. A 0.3 M aq. NaOH stock solution was prepared by adding NaOH (600 mg, 15 mmol) to a 50 mL volumetric flask followed by DI H_2O. $PAA_{P\&G}$ (250 mg) was added to separate 20 mL vials equipped with stir bars followed by aq. NaOH (0.3 M, 5.0 mL). The vials were stirred at 350 rpm on a hot plate at 80 °C for the appropriate time (i.e., 1, 2, 12, 15, 18, and 25 h). Then, the reaction mixture was cooled to rt in a water bath at 25 °C followed by adding acetic acid (90 μL, 1.5 mmol) to quench the NaOH. A pH of 6–7 was observed using pH paper.

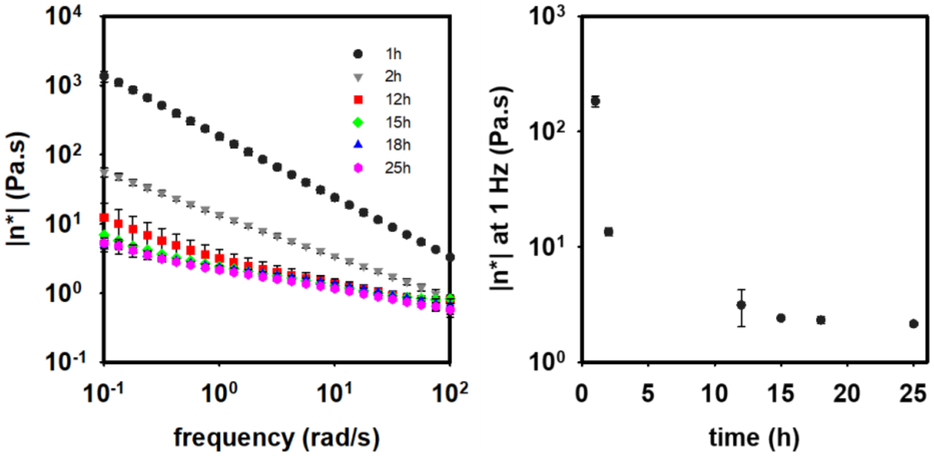

Supplementary Fig. 1. Plot of complex viscosity versus frequency (left) and complex viscosity at 1 Hz versus time (right) for decrosslinking $PAA_{P\&G}$ (5% w/v) using 0.3 M aq. NaOH at 80 °C. Error bars are derived from an average of 2 runs.

Monitoring decrosslinking with 0.3 M aq. H_2SO_4.

A 0.8 M aq. H_2SO_4 stock solution was prepared by adding H_2SO_4 (2.15 mL, 4.0 mmol) to a 50 mL volumetric flask followed by DI water. $PAA_{P\&G}$ (250 mg) and aq. H_2SO_4 (0.8 M, 5.0 mL) were added to a 15 mL pressure vessel equipped with a stir bar. After sealing the vessel, the reaction mixture was heated at 120 °C for the appropriate time (i.e., 1, 2.5, 11.5, 14, and 24 h). Then, the reaction mixture was cooled to rt in a water bath at 25 °C and quenched with aq. Na_2CO_3 (2 mL, 2 M). A pH of approximately 3 was observed using pH paper.

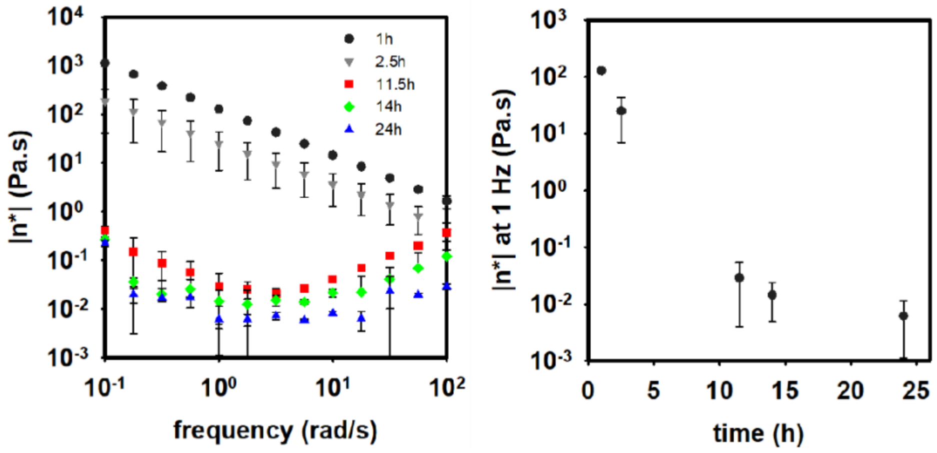

Supplementary Fig. 2. Plot of complex viscosity versus frequency (left) and complex viscosity at 1Hz versus time (right) for decrosslinking $PAA_{P\&G}$ (5% w/v) using 0.8 M aq. H_2SO_4 at 120 °C. Error bars are derived from an average of 2 runs.

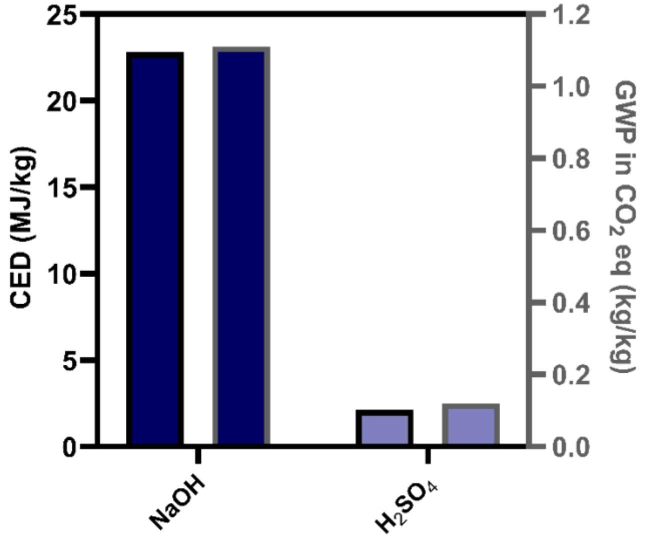

Supplementary Fig. 3. Comparing the cumulative energy demand and global warming potential for sulfuric acid versus sodium hydroxide using data from the SimaPro database.

Decrosslinking $PAA_{P\&G}$ for chain-shortening experiments

A 0.8 M aq. H_2SO_4 solution was prepared by adding H_2SO_4 (6.84 mL, 128 mmol, 1.5 equiv) to a 350 mL pressure vessel containing DI H_2O (160 mL) stirring at 350 rpm. Thereafter, $PAA_{P\&G}$ (8,000 mg, 85.1 mmol, 1 equiv) was added. The vessel was sealed and the reaction stirred at 120 °C for 24 h. The resulting decrosslinked polymer was used for the subsequent sonication experiments (see chain-shortening sections).

IV. Evaluating polymer recovery and chemical structure after sonication

A portion of the decrosslinked $PAA_{P\&G}$ solution (25 mL, 2.5 wt%) and DI H_2O (25 mL) were poured into a jacketed beaker equipped with a stir bar. While flowing cold water through the jacket, the decrosslinked $PAA_{P\&G}$ was sonicated at 100% amplitude (280 W) for 1 min. During sonication, the temperature rose from 10–15 °C to 50 °C. 940 mg (97%) was recovered after dialyzing and freeze-drying (see general experimental).

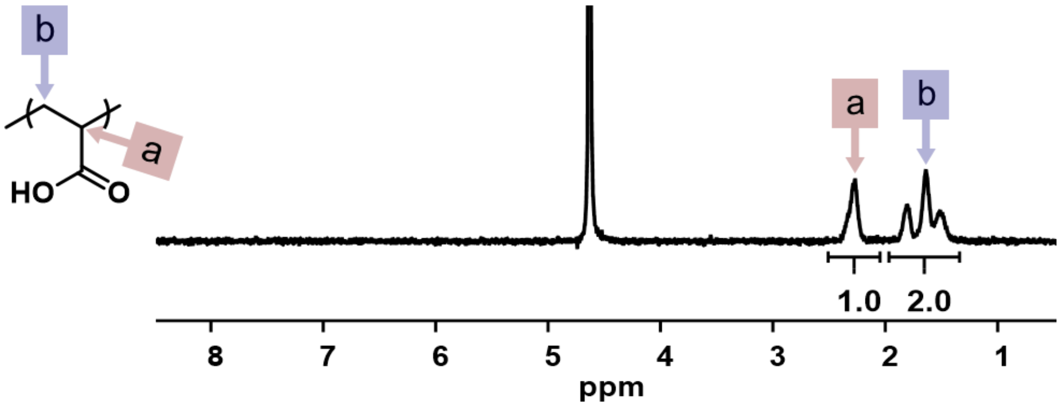

Supplementary Fig. 4. ^1H NMR spectra of sonicated $PAA_{P\&G}$ (500 MHz, D_2O).

V. Monitoring chain-shortening and energy consumption at 2.5 and 5.0% w/v

Monitoring chain-shortening over time of decrosslinked $PAA_{P\&G}$ at 5.0% w/v

Two portions of the decrosslinked $PAA_{P\&G}$ solution (50 mL each) were poured into jacketed beakers equipped with a stir bar. While flowing cold water through the jacket, the decrosslinked $PAA_{P\&G}$ was sonicated at 100% amplitude (290 W) while collecting 1.0 mL aliquots at 1, 2, 5, 10, and 15 min. During sonication, the temperature rose from 10–15 °C to 50 °C. The aliquots were quenched using aq. Na_2CO_3 (2 M, 0.4 mL). The aliquots were diluted (to 1–1.5 mg/mL) with 0.2 M aq. $NaNO_3$/ethylene glycol (99:1 v/v) and analyzed via SEC.

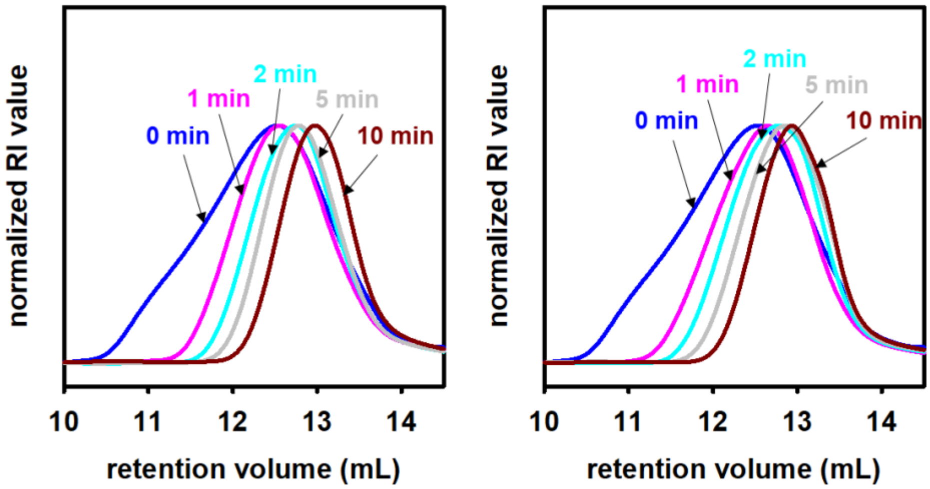

Supplementary Fig. 5. SEC traces for the chain-shortening of decrosslinked $PAA_{P\&G}$ at 5.0% w/v.

Supplementary Table 1. Maximum power (P_{max}) consumed during sonication for $PAA_{P\&G}$ at 5% w/v.

[PAA] (w/v %)	mass (mg)	mmol	run 1 P_{max} (W)	run 2 P_{max} (W)
5	2,500	26.6	290	290

Maximum specific energy (w_{max}) values were determined using supplimentary equation (1)

Supplementary Table 2. Weight average molar mass (M_w), dispersity (Đ) and specific energy (w_{max}) data for sonications of decrosslinked $PAA_{P\&G}$ at 5.0% w/v.

time (min)	run 1			run 2		
	M_w (kg/mol)	Đ	w_{max}	M_w (kg/mol)	Đ	w_{max}
0	870	2.8	0			
1	460	1.8	7	430	1.7	7
2	340	1.6	14	320	1.6	14
5	270	1.3	35	270	1.4	35
10	220	1.3	70	200	1.4	70

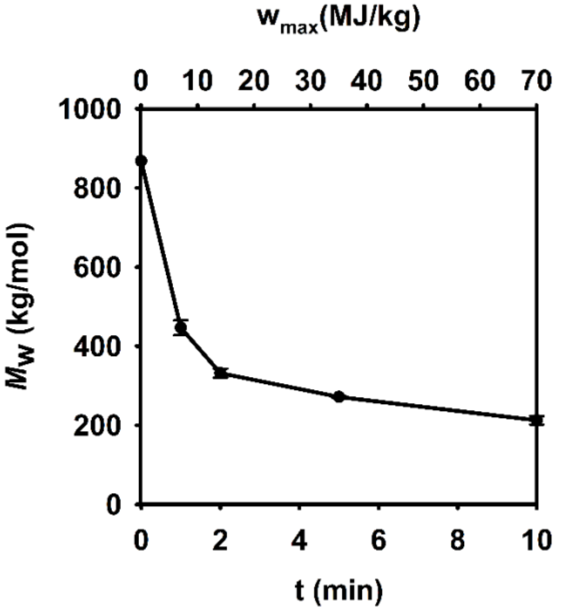

Supplementary Fig. 6. Weight average molar mass (M_w) versus time (t) and maximum specific energy (w_{max}) plot for $PAA_{P\&G}$ sonication at 5% w/v. Error bars are derived from an average of 2 runs.

Monitoring chain-shortening over time of decrosslinked $PAA_{P\&G}$ at 2.5% w/v

A portion of the decrosslinked $PAA_{P\&G}$ solution (50 mL) was diluted to 100 mL using DI water to make 2.5% w/v solution. This solution was poured into two jacketed beakers (50 mL each) equipped with a stir bar. While flowing cold water through the jacket, the decrosslinked $PAA_{P\&G}$ was sonicated at 100% amplitude (280 W) while collecting 1.0 mL aliquots at 0, 0.5, 1, 2, 5, and 10 min. During sonication, the temperature rose from 10–15 °C to 50 °C. The aliquots were quenched using aq. Na_2CO_3 (2 M, 0.2 mL). The aliquots were diluted (to 1–1.5 mg/mL) with 0.2 M aq. $NaNO_3$/ethylene glycol (99:1 v/v) and analyzed via SEC.

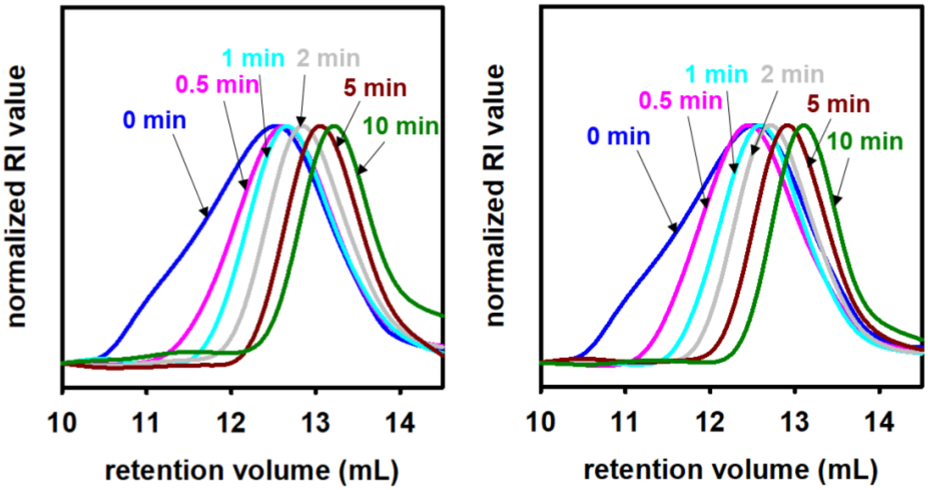

Supplementary Fig. 7. SEC traces for the chain-shortening of decrosslinked $PAA_{P\&G}$ at 2.5% w/v

Supplementary Table 3. Maximum power (P_{max}) consumed during sonication for $PAA_{P\&G}$ at 2.5% w/v.

[PAA] (w/v %)	mass (mg)	mmol	run 1 P_{max} (W)	run 2 P_{max} (W)
2.5	1,250	13.3	280	280

Maximum specific energy (w_{max}) values were determined using supplementary equation (1)

Supplementary Table 4. Weight average molar mass (M_w), dispersity (Đ) and specific energy (w_{max}) data for sonications of decrosslinked $PAA_{P\&G}$ at 2.5% w/v.

time (min)	run 1			run 2		
	M_w (kg/mol)	Đ	w_{max}	M_w (kg/mol)	Đ	w_{max}
0	870	2.8	0	870	2.8	0
0.5	450	1.9	6.7	560	2.0	6.7
1	330	1.7	13	390	1.6	13
2	240	1.5	27	290	1.5	27
5	180	1.4	67	210	1.4	67
10	140	1.3	130	160	1.4	130

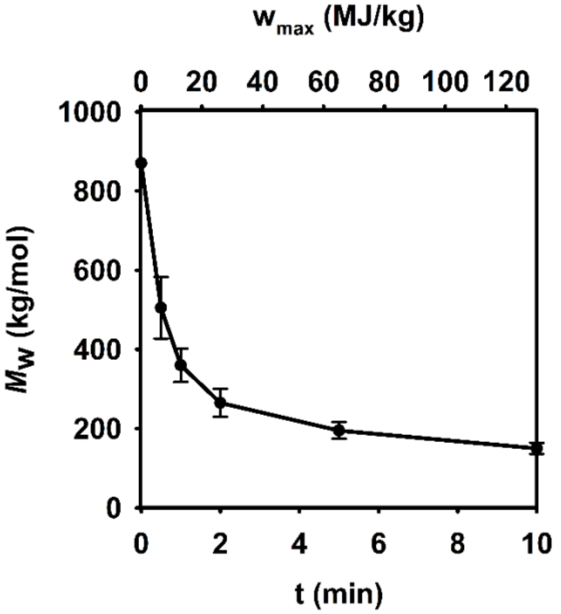

Supplementary Fig. 8. Weight average molar mass (M_w) versus time (t) and maximum specific energy (w_{max}) plot for $PAA_{P\&G}$ sonication at 2.5% w/v. Error bars are derived from an average of 2 runs.

VI. Fischer esterification studies

Commercial PAAs (i.e., PAA_{SIGMA1} and PAA_{SIGMA2}) are low molecular weight (< 450 kg/mol) relative to the chain-shortened $PAA_{P\&G}$. Consequently, shorter esterification times are needed for commercial PAAs (4 h) relative to the chain-shortened materials (10 h).

Effect of alcohol equivalents on conversion

Reactions were run under identical conditions except for the amounts of 2-ethylhexanol (2-EHOH) (3, 5, 10, 15 equiv.) used relative to PAA_{SIGMA2}. 2-EHOH (2.60 mL, 16.7 mmol, 3.00 equiv.; 4.30 mL, 27.8 mmol, 5.00 equiv.; 8.70 mL, 55.5 mmol, 10.0 equiv.; 13.0 mL, 83.3 mmol, 15.0 equiv.) was added to seperate 20 mL vials equipped with stir bars. *p*-TsOH (527 mg, 2.80 mmol, 0.500 equiv.) was added to each vial and stirred until dissolved. The vials were subsequently heated to 120 °C, then PAA_{SIGMA2} (400 mg, 5.60 mmol, 1.0 equiv.) was added. The vials were capped and stirred for 4 h at 120 °C. The initially heterogeneous reaction mixture becomes homogenous over time (see Figure S9).

Thereafter, the vials were cooled to rt in a water bath. The poly(2-ethylhexyl acrylate)$_{SIGMA2}$ (P(2-EHA))$_{SIGMA2}$) was isolated by precipitating into MeOH (10 mL) and removing the supernatent. Then, the polymer was purified by dissolving in minimal amounts of THF (1 mL), precipitating into MeOH (10 mL), and removing the supernatent. This process was repeated three times. The resulting solid was dried under high vacuum at 60 °C for 3 h.

The isolated yields were 77% (3 equiv), 77% (5 equiv), 89% (10 equiv), and 89% (15 equiv).

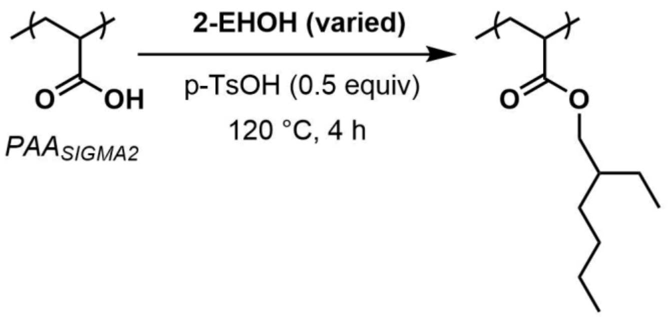

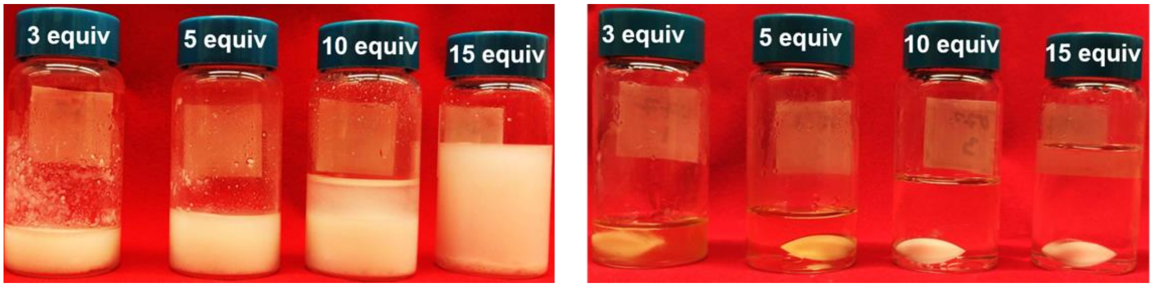

Supplementary Fig. 9. Esterification reactions for 3–15 equiv. 2-ethylhexanol before (left) and after (right) heating to 120 °C for 4 h.

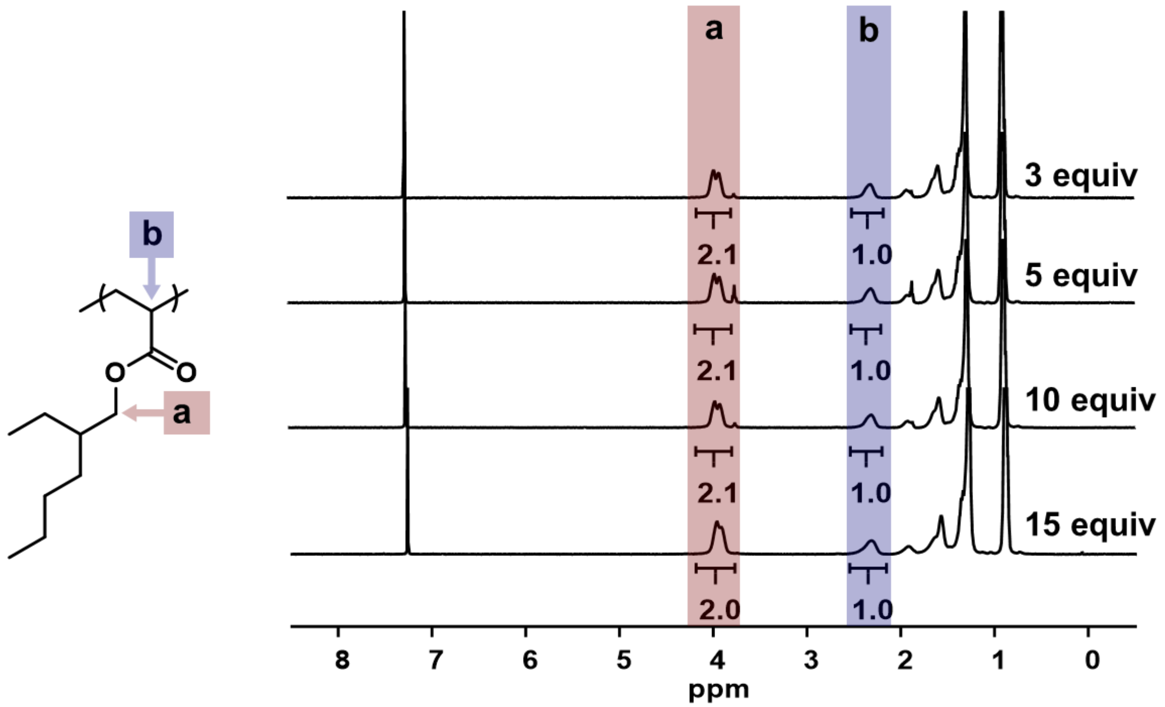

Supplementary Fig. 10. ^{1}H NMR spectra of P(2-EHA)$_{SIGMA2}$ made from 3–15 equiv. alcohol (500 MHz, $CDCl_3$).

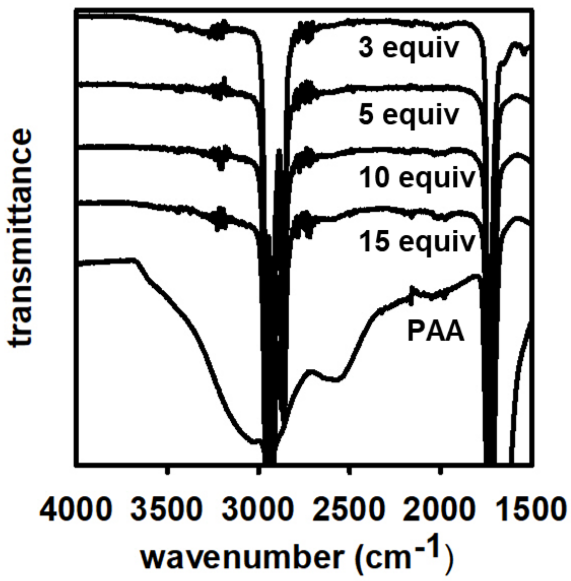

Supplementary Fig. 11. IR spectra of P(2-EHA)$_{SIGMA2}$ made from 3–15 equiv. alcohol.

Effect of adding water on conversion
2-ethylhexanol (1.95 mL, 12.49 mmol, 3.00 equiv) was added to 15 mL pressure vessels equipped with stir bars, followed by DI H_2O (0.22 mL, 12.49 mmol, 3.00 equiv). Sulfuric acid (0.055 mL, 1.04 mmol, 0.25 equiv) was then added and the vessel was stirred at 120 °C. PAA_{SPP} (300 mg, 4.20 mmol, 1.00 equiv) was subsequently added and the pressure vessels were immediately sealed and left to run for 6 h. Thereafter, the vials were placed in a water bath to cool and then the polymer was precipitated with MeOH (~10 mL). The $P(2\text{-EHA})_{SPP}$ obtained was purified by dissolving in minimal amounts of THF (~2 mL) and precipitating with MeOH (~10 mL) twice, followed by drying under high vacuum at 60 °C for 3 h.

In the replication experiment, the isolated yield was 79%.

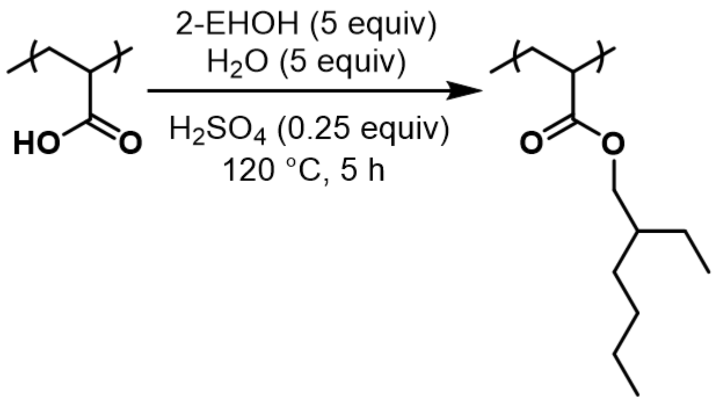

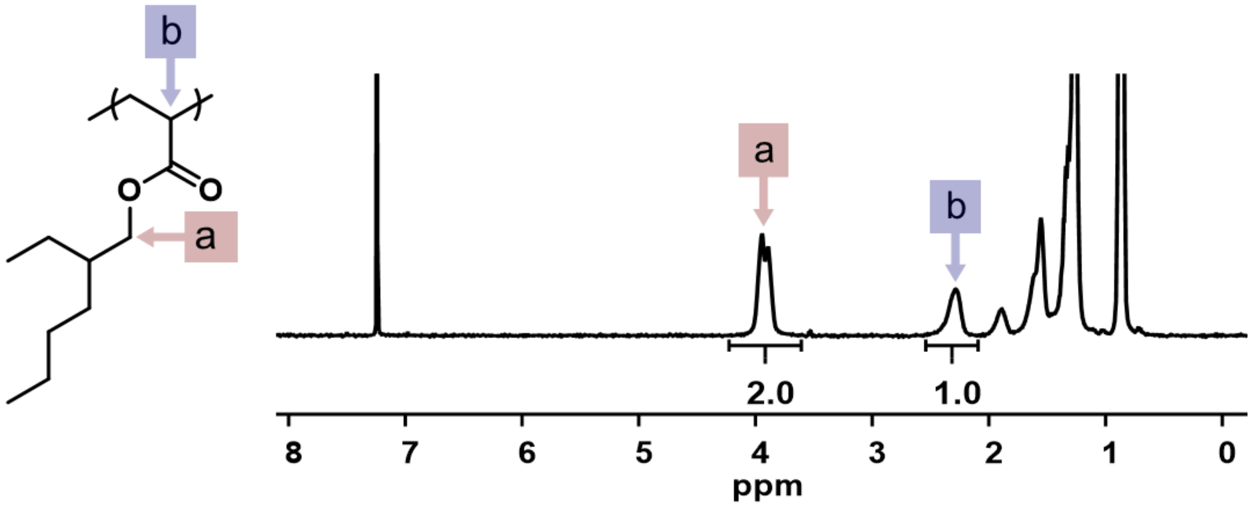

Supplementary Fig. 12. ^1H NMR spectra of $P(2\text{-EHA})_{SPP}$ (500 MHz, $CDCl_3$).

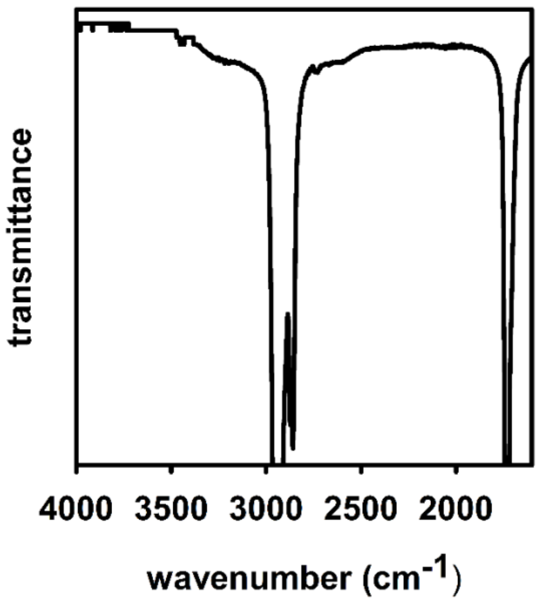

Supplementary Fig. 13. IR spectrum of P(2-EHA)$_{SPP}$ made in the presence of added H_2O

Effect of adding water on conversion for small molecule carboxylic acids

(This experiment was run in duplicate.) To two 15 mL pressure vessels equipped with stir bars, 2-EHOH (3.91 mL, 25 mmol, 5.0 equiv.), sulfuric acid (0.067 mL, 1.25 mmol, 0.25 equiv.), and acetic acid (0.29 mL , 5.0 mmol, 1.0 equiv.) were added. Then, DI H_2O (0.45 mL, 25 mmol, 5.00 equiv.) was added to one vessel. Both vessels were sealed and stirred at 120 °C for 8 h. Thereafter, the vessels were cooled in a rt water bath and aliquots (0.1 mL) were diluted with 2:1 $CDCl_3$/pyridine-d_5 (0.4 mL) for ^{1}H NMR spectroscopic analysis.

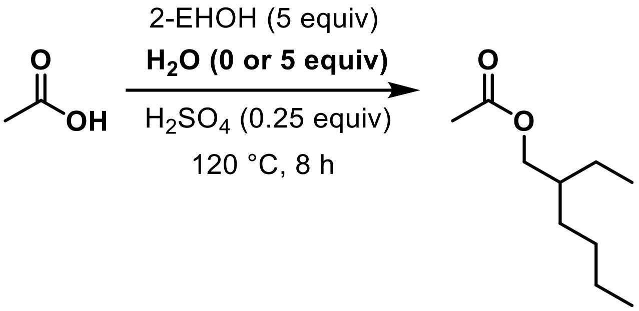

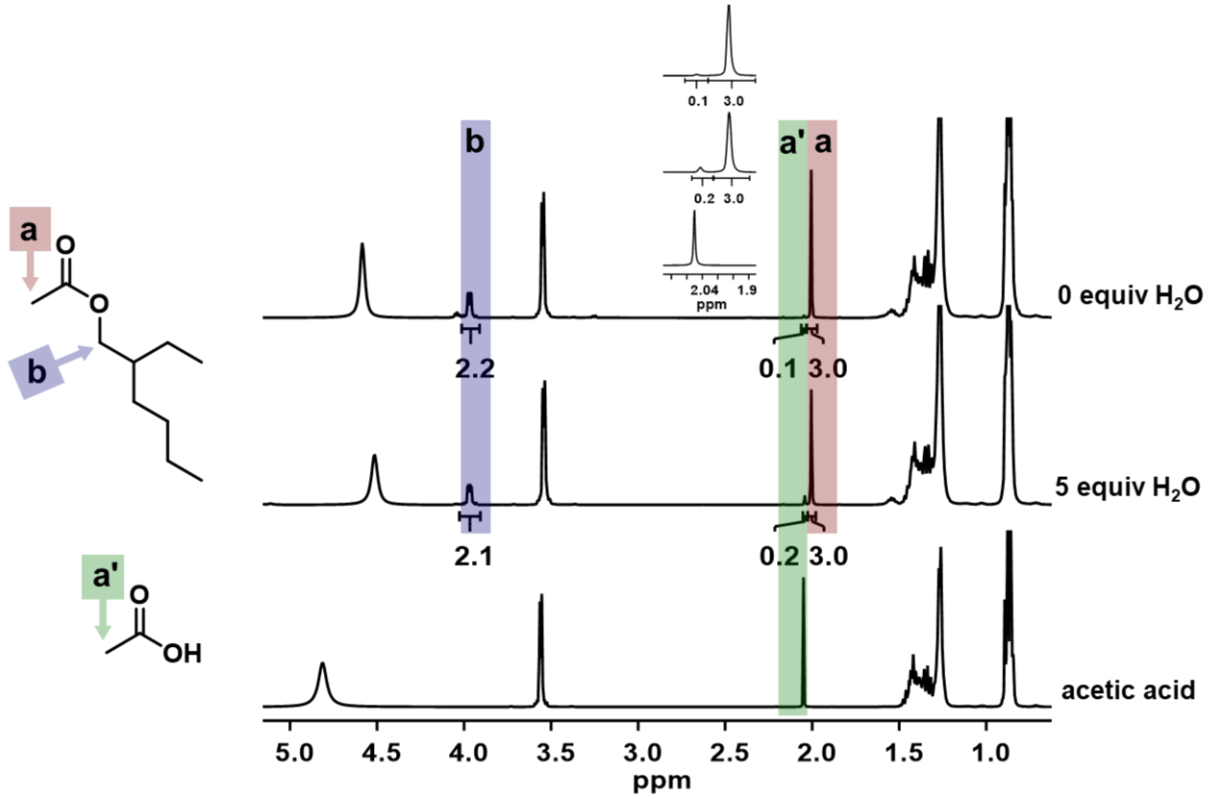

Supplementary Fig. 14. ^{1}H NMR spectra of acetic acid esterification with 2-ethylhexanol in the presence (middle) and absence (top) of water (500 MHz, $CDCl_3$/pyridine-d_5 at 2:1).

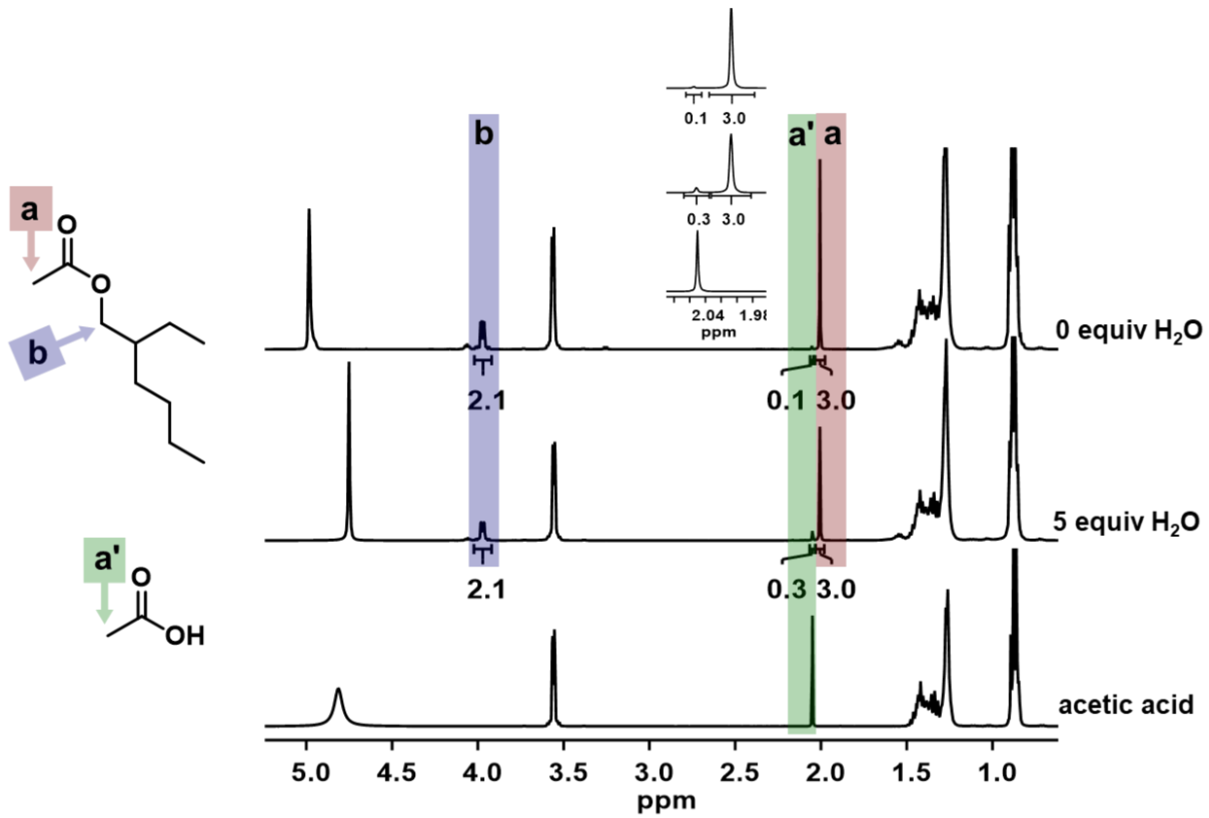

Supplementary Fig. 15. ^{1}H NMR spectra of acetic acid esterification with 2-ethylhexanol in the presence (middle) and absence (top) of water (500 MHz, $CDCl_3$/pyridine-d_5 at 2:1).

Supplementary Table 5. Calculated conversions for H_2O (0 or 5 equiv) esterification conditions

	a	a'	% conversion
H_2O (0 equiv)	0.1	3.0	97%
H_2O (5 equiv)	0.2	3.0	94%

	average
H_2O (0 equiv)	97%
H_2O (5 equiv)	94%

	a	a'	% conversion
H_2O (0 equiv)	0.1	3.0	97%
H_2O (5 equiv)	0.2	3.0	94%

(This experiment was run in duplicate.) To two 15 mL pressure vessels equipped with stir bars, EtOH (1.5 mL, 26 mmol, 5.1 equiv.), H_2SO_4 (0.0670 mL, 1.25 mmol, 0.245 equiv.) and acetic acid (0.29 mL, 5.1 mmol, 1.0 equiv.) were added. Then, DI H_2O (0.45 mL, 25 mmol, 4.9 equiv.) was added to one vessel. Both vessels were sealed and stirred at 120 °C for 8 h. Thereafter, the vessels were cooled to rt in a water bath and aliquots (0.1 mL) were diluted with 2:1 $CDCl_3$/pyridine-d_5 (0.4 mL) for ^1H NMR spectroscopic analysis.

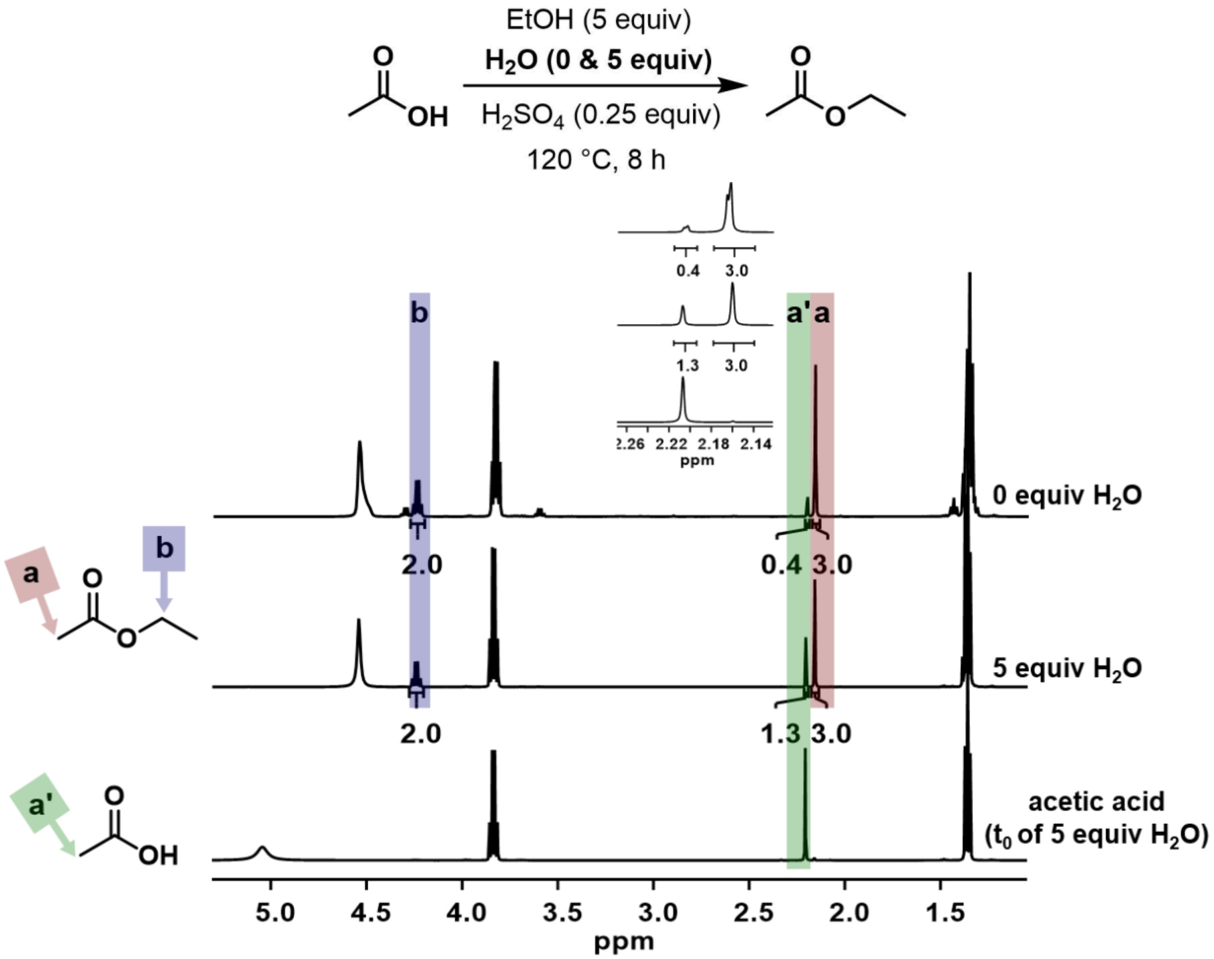

Supplementary Fig. 16. ^1H NMR spectra of acetic acid esterification with EtOH in the presence (middle) and absence (top) of water (500 MHz, $CDCl_3$/pyridine-d_5 at 2:1).

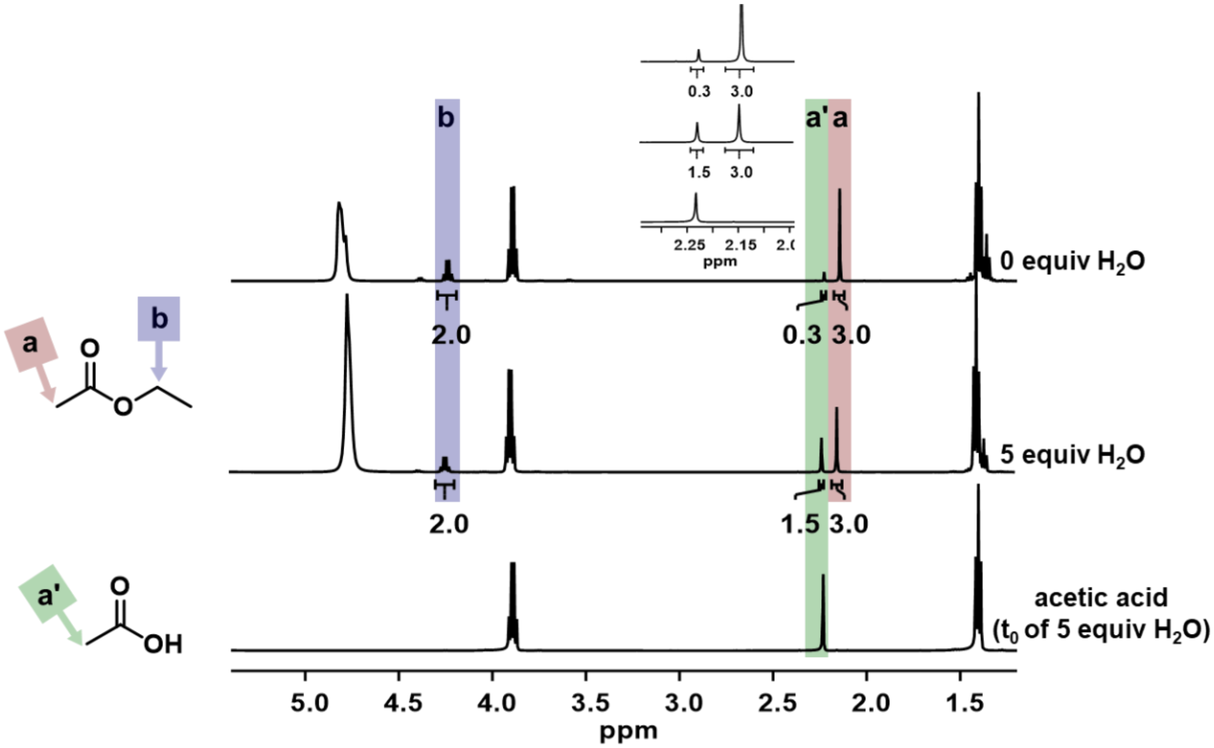

Supplementary Fig. 17. ^{1}H NMR spectra of acetic acid esterification with EtOH in the presence (middle) and absence (top) of water (500 MHz, $CDCl_3$/pyridine-d_5 at 2:1).

Supplementary Table 6. Calculated conversions for H_2O (0 or 5 equiv) esterification conditions

	a'	a	% conversion
H_2O (0 equiv)	0.4	3.0	88%
H_2O (5 equiv)	1.3	3.0	70%

	average
H_2O (0 equiv)	90%
H_2O (5 equiv)	68%

	a'	a	% conversion
H_2O (0 equiv)	0.3	3.0	91%
H_2O (5 equiv)	1.5	3.0	67%

To two 15 mL pressure vessels equipped with stir bars, 2-EHOH (1.8 mL, 12. mmol, 5.0 equiv.), H_2SO_4 (0.031 mL, 0.58 mmol, 0.25 equiv.) and undecanoic acid (433 mg, 2.30 mmol, 1.00 equiv.) were added. Then, DI H_2O (0.21 mL, 12 mmol, 5.0 equiv.) was added to one vessel. Both vessels were sealed and stirred at 120 °C for 8 h. Thereafter, the vessels were cooled in a rt water bath and aliquots (0.1 mL) were diluted with 2:1 $CDCl_3$/pyridine-d_5 (0.4 mL) for ^1H NMR spectroscopic analysis.

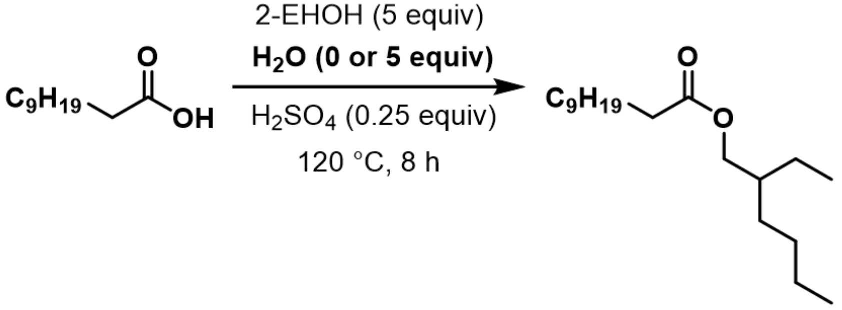

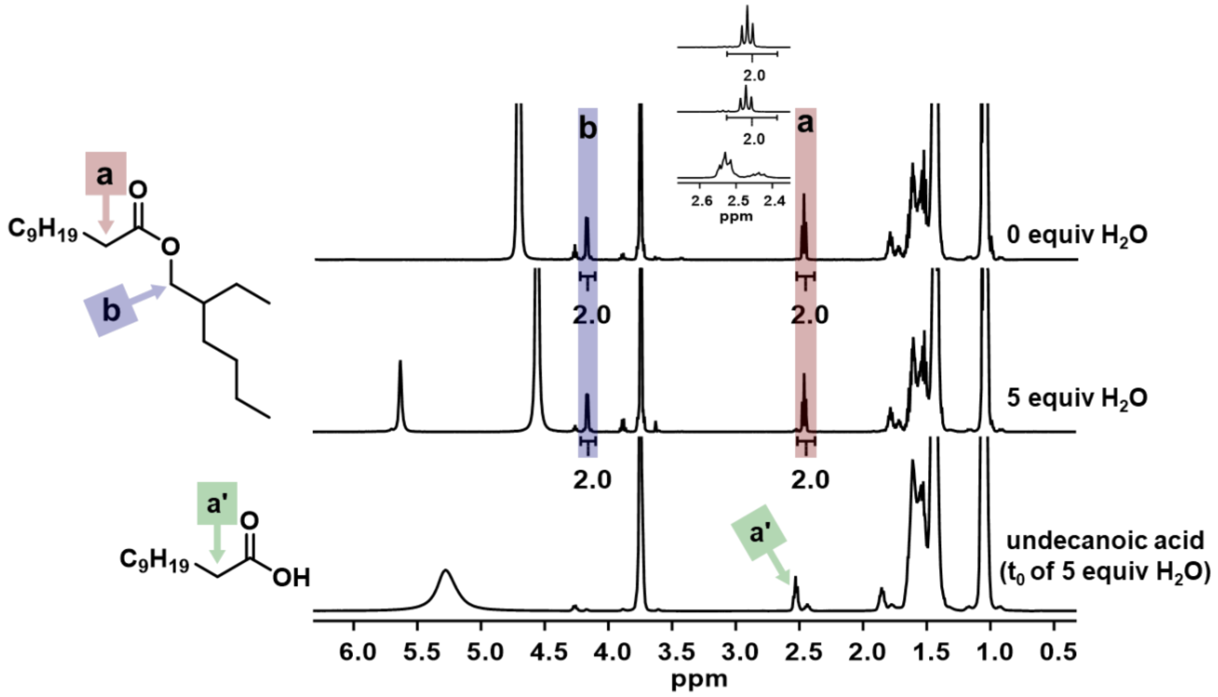

Supplementary Fig. 18. ^1H NMR spectra of undecanoic acid esterification with 2-ethylhexanol in the presence (middle) and absence (top) of water (500 MHz, $CDCl_3$/pyridine-d_5 at 2:1).

To two 15 mL pressure vessels equipped with stir bars, 2-EHOH (1.8 mL, 12 mmol, 5.2 equiv.), H_2SO_4 (0.031 mL, 0.58 mmol, 0.25 equiv.) and decanoic acid (400 mg, 2.32 mmol, 1.00 equiv.) were added. Then, DI H_2O (0.21 mL, 12 mmol, 5.2 equiv.) was added to one vessel. Both vessels were sealed and stirred at 120 °C for 8 h. Thereafter, the vessels were cooled to rt in a water bath and aliquots (0.1 mL) were diluted with 2:1 $CDCl_3$/pyridine-d_5 (0.4 mL) for ^1H NMR spectroscopic analysis.

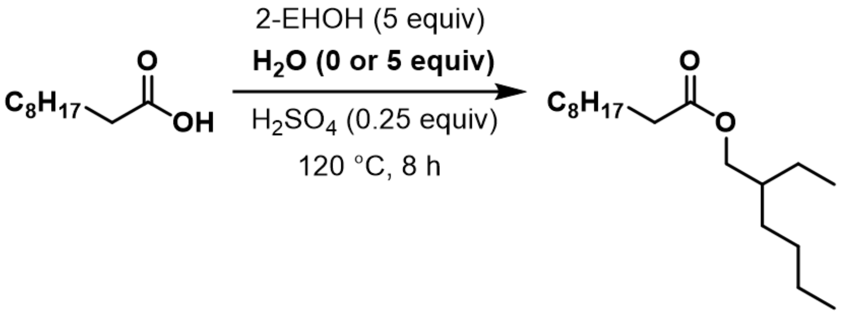

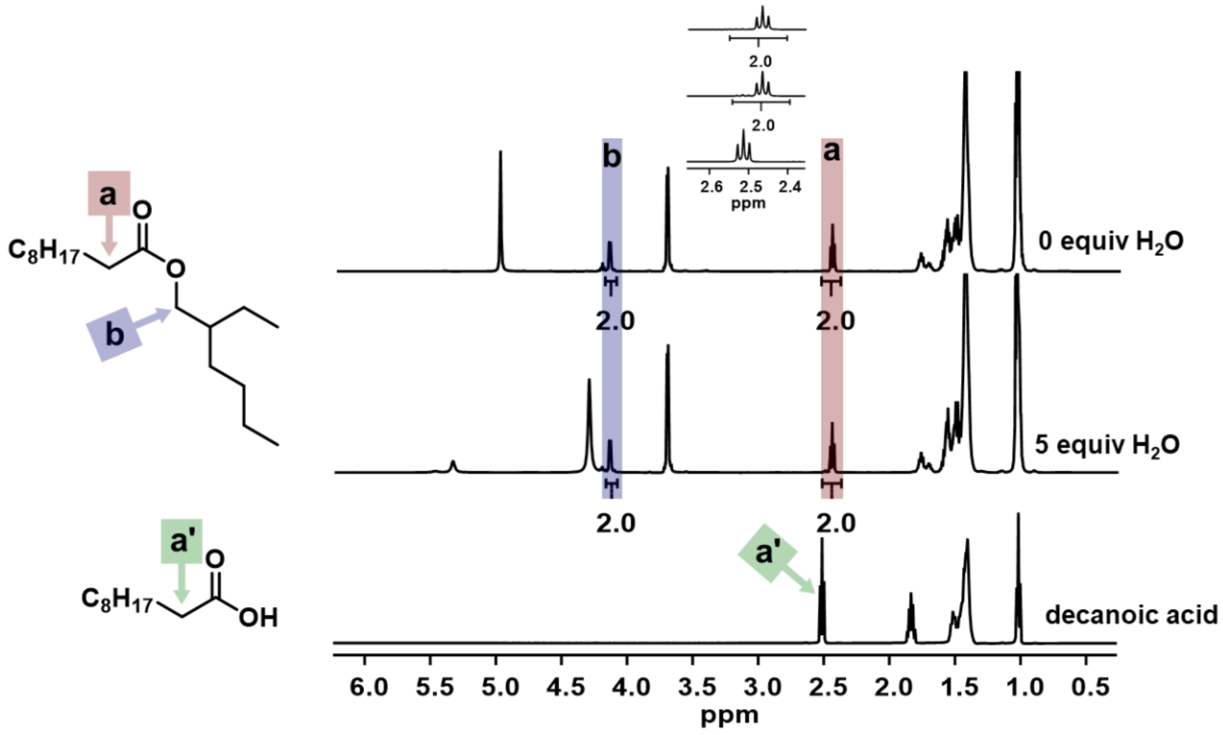

Supplementary Fig. 19. ^1H NMR spectra of decanoic acid esterification with 2-ethylhexanol in the presence (middle) and absence (top) of water (500 MHz, $CDCl_3$/pyridine-d_5 at 2:1).

Supplementary Table 7. Calculated conversions for H_2O (0 or 5 equiv) esterification conditions

	a'	a	% conversion
H_2O (0 equiv)	0	2.0	100%
H_2O (5 equiv)	0	2.0	100%

	average
H_2O (0 equiv)	100%
H_2O (5 equiv)	100%

	a'	a	% conversion
H_2O (0 equiv)	0	2.0	100%
H_2O (5 equiv)	0	2.0	100%

To two 15 mL pressure vessels equipped with stir bars, EtOH (0.58 mL, 9.98 mmol, 5.0 equiv), sulfuric acid (0.027 mL, 0.50 mmol, 0.25 equiv) and undecanoic acid (400 mg , 2 mmol, 1.0 equiv) were added. Then, DI H_2O (0.18 mL , 9.98 mmol, 5.0 equiv) was added to one vessel. Both vessels were sealed stirred at 120 °C for 8 h. Thereafter, the vessels were cooled to rt in a water bath and aliquots (0.1 mL) were diluted with 2:1 $CDCl_3$/pyridine-d_5 (0.4 mL) for ^1H NMR spectroscopic analysis.

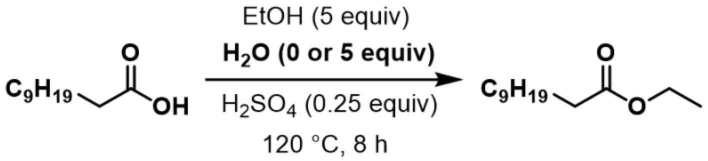

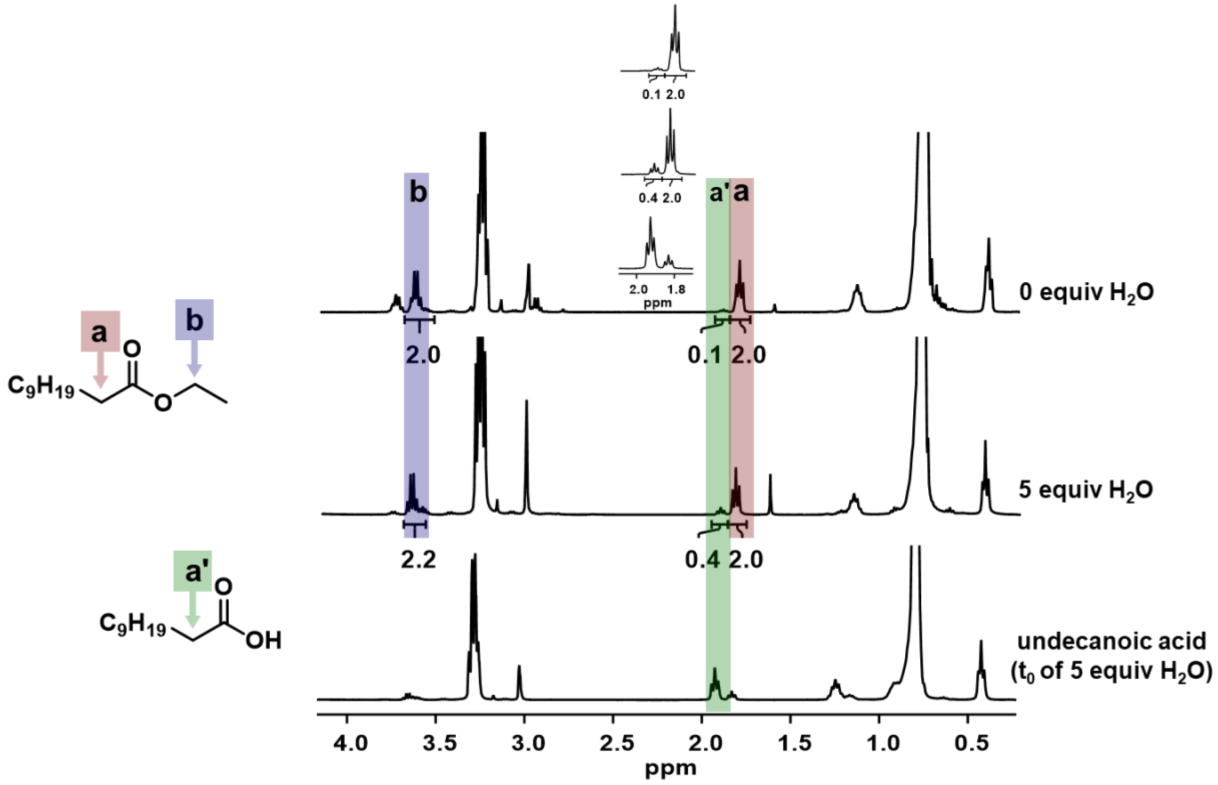

Supplementary Fig. 20. ^1H NMR spectra of undecanoic acid esterification with EtOH in the presence (middle) and absence (top) of water (500 MHz, $CDCl_3$/pyridine-d_5 at 2:1).

To two 15 mL pressure vessels equipped with stir bars, EtOH (0.74 mL, 12.6 mmol, 5.0 equiv), sulfuric acid (0.034 mL, 0.63 mmol, 0.25 equiv), and undecanoic acid (470 mg , 2.52 mmol, 1.0 equiv) were added. Then, DI H_2O (0.23 mL , 12.6 mmol, 5.0 equiv) was added to one vessel. Both vessels were sealed and stirred at 120 °C for 8 h. Thereafter, the vessels were cooled to rt in a water bath and aliquots (0.1 mL) were diluted with 2:1 $CDCl_3$/pyridine-d_5 (0.4 mL) for ^1H NMR spectroscopic analysis.

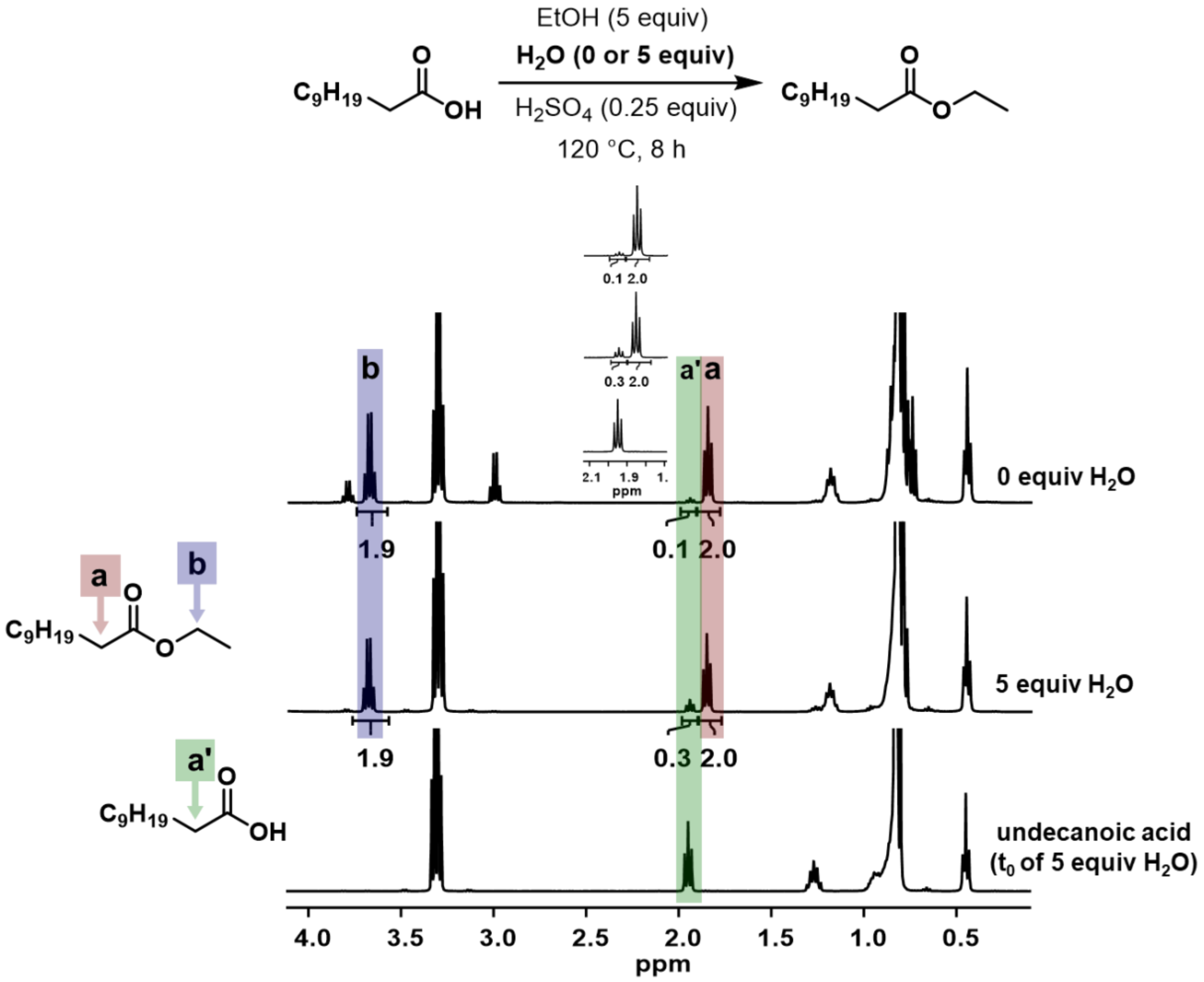

Supplementary Fig. 21. ^1H NMR spectra of undecanoic acid esterification with EtOH in the presence (middle) and absence (top) of water (500 MHz, $CDCl_3$/pyridine-d_5 at 2:1).

Supplementary Table 8. Calculated conversions for H_2O (0 or 5 equiv) esterification conditions

	a'	a	% conversion
H_2O (0 equiv)	0.1	2.0	95%
H_2O (5 equiv)	0.4	2.0	83%

	average
H_2O (0 equiv)	95%
H_2O (5 equiv)	85%

	a'	a	% conversion
H_2O (0 equiv)	0.1	2.0	95%
H_2O (5 equiv)	0.3	2.0	87%

VII. Free-energy calculations

Background on free energy calculations

The calculation of free-energy differences between two states is a common and widely adopted method in computational chemistry.[2] To assess the difference between two states, the states must have a configurational overlap large enough for a comparison to be made. In practice, most end states do not have such an overlap, necessitating the use of bridge states that are a mix of both systems of interest. Herein the degree of perturbation is denoted as λ.

System construction

Nonamers AA_9, BA_8AA_1, AA_8BA_1, and BA_9 were constructed using Avogadro[3] and then solvated in a 3:1 butanol:water cuboid using PACKMOL,[4] providing a 12 Å buffer between the nonamer and the edge of the cuboid. This resulted in a 41.841 x 44.981 x 45.167 Å box with 480 butanols and 160 waters for BA_8AA_1 and BA_9, and a 37.678 x 40.876 x 35.483 Å box with 333 butanols and 111 waters for AA_9 and AA_8BA_1. All of the nonamers studied were isotactic. TIP3P parameters[5] were used for water, and parameters for butanol and the nonamers were derived from CGenFF[6] using MATCH.[7]

Molecular Dynamics

Molecular dynamics studies were performed using the CHARMM molecular mechanics platform (developmental version 44a1)[8] with the domain decomposition (DOMDEC) computational kernels on graphics processing units (GPUs).[9] Molecular dynamics were performed using the canonical ensemble (NVT) at 298.15 K using a Langevin thermostat. The Leapfrog Verlet integrator was used with an integration time of 2 fs. Electrostatic interactions were modeled using a particle-mesh Ewald method[10,11,12] with a grid spacing of 1 Å, interpolation order of 6, and a κ-value of 0.32 $Å^{-1}$. Van der Waals interactions were modelled using a 9 Å switching radius, 10 Å cutoff radius, and a 12 Å neighbor list.

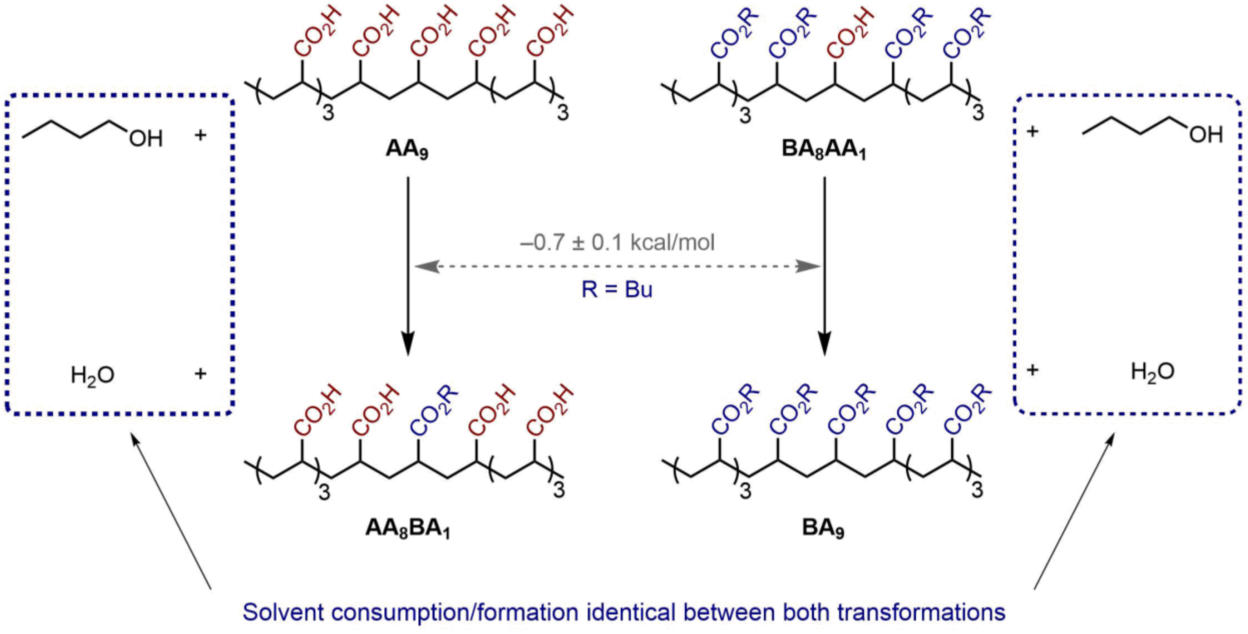

Supplementary Fig. 22. The full thermodynamic cycle used to evaluate the free energy of esterification.

Calculating the difference in free energy of esterification (ΔΔA)

The difference in free energy of esterification (ΔΔA) was calculated using the Multistate Bennet Acceptance Ratio method[13] using a dual topology approach. Both AA_9 and BA_8AA_1 were perturbed to AA_8BA_1 and BA_9, respectively, using 11 discrete λ states, 0 → 1, in steps of Δλ = 0.1. Perturbation of λ was achieved using the block module of CHARMM, λ values held constant using the MSλD *ffix* keyword.[14] Non-bonding interactions were scaled by λ using a soft-core potential.[15] Prior to molecular dynamics simulations, a system was subjected to 200 steps of steepest descent minimization. Each λ state was subjected to 200 steps of steepest descent minimization, followed by equilibration for 5 ns. Production runs consisted of 50 ns of simulation, with trajectory frames saved every 2,500 timesteps (yielding 10,000 frames total).

Energy Calculation Results

The free energy difference between the λ=0 and other lambda states (0.1 to 1.0) for the AA_9 and BA_8AA_1 systems are shown in Table S5. From the ΔA value for when λ=1 for both systems, the ΔΔA of esterification is calculated to be –0.7 ± 0.1 kcal/mol. As the consumption of butanol and the evolution of water is expected to be identical in AA_8BA_1 and BA_9, the ΔA of butanol consumption and water formation during the process of esterification was ignored, as those terms would cancel out in the calculation of ΔΔA of esterification (Figure S22).

Supplementary Table 9. Values for the difference in free energy between λ=0 and other λ values for the AA_9 and BA_8AA_1 systems.

λ	$AA_9 \rightarrow AA_8BA_1$ ΔA relative to λ=0		$BA_8AA_1 \rightarrow BA_9$ ΔA relative to λ=0	
	in k_BT	in kcal/mol	in k_BT	in kcal/mol
0.0	0.0 ± 0.0	0.0 ± 0.0	0.0 ± 0.0	0.0 ± 0.0
0.1	22.5 ± 0.1	13.35 ± 0.07	21.2 ± 0.1	12.58 ± 0.06
0.2	36.8 ± 0.1	21.78 ± 0.08	35.1 ± 0.1	20.74 ± 0.06
0.3	48.4 ± 0.1	28.70 ± 0.08	46.4 ± 0.1	27.51 ± 0.06
0.4	58.5 ± 0.1	34.65 ± 0.08	56.4 ± 0.1	33.44 ± 0.06
0.5	67.4 ± 0.1	39.92 ± 0.08	65.4 ± 0.1	38.75 ± 0.06
0.6	75.6 ± 0.1	44.76 ± 0.08	73.7 ± 0.1	43.66 ± 0.06
0.7	83.1 ± 0.1	49.24 ± 0.08	81.4 ± 0.1	48.21 ± 0.06
0.8	90.0 ± 0.1	53.31 ± 0.08	88.4 ± 0.1	52.37 ± 0.06
0.9	96.1 ± 0.1	56.93 ± 0.08	94.7 ± 0.1	56.10 ± 0.06
1.0	101.3 ± 0.1	60.04 ± 0.08	100.2 ± 0.1	59.35 ± 0.06

Comparing the difference in free energy of esterification between poly(acrylic acid) and poly(butyl acrylate) with the effects of changing solvent composition.

The free energy of a given reaction is dependent on the free energy of the reaction at standard conditions (calculated when the concentration of both products and reactants is 1M), and concentration of reagents in the given conditions (supplementary equation (2)).

$$\Delta A = \Delta A^{\circ} + k_B T \ln\left(\frac{[\text{Products}]}{[\text{Reactants}]}\right) \quad \text{Supplementary Equation (2)}$$

In an esterification reaction, the concentration of water increases over time, and the concentration of alcohol decreases. Using the right-hand term of supplementary equation (2), we can estimate the effect of changing solvent composition on the free energy of the reaction (supplementary equation (3)).

$$\Delta\Delta A_{\text{solv}} = k_B T\left(\ln\left(\frac{[\text{Water}]_{\text{end}}}{[\text{Alcohol}]_{\text{end}}}\right) - \ln\left(\frac{[\text{Water}]_{\text{Start}}}{[\text{Alcohol}]_{\text{Start}}}\right)\right) \quad \text{Supplementary Equation (3)}$$

Using the starting conditions of the reaction, and the expected conditions if full conversion is achieved (1 unit of alcohol is replaced by 1 unit of water), we can estimate the energetic effect that the change in solvent composition has on ΔA, denoted as $\Delta\Delta A_{solv}$ (Table S6.)

Supplementary Table 10. Expected $\Delta\Delta A_{solv}$ values for select reaction conditions.

Scenario	Alcohol : Water at start	Alcohol : Water at end	$\Delta\Delta A_{solv}$ in units of k_BT
Figure S21, esterification conditions	5:3	4:4	0.51
Figure S21, small molecule testing	5:5	4:6	0.41
Free energy calculation	3:1	2:2	1.10

In all of the scenarios listed in Table S6, the $\Delta\Delta A_{solv}$ is less than or comparable in magnitude to the change in free energy of esterification due the change in hydrophobicity (-1.16 units of k_BT). This shows that the increase in hydrophobicity as esterification progresses counters the free energy contribution due to buildup of water, allowing the reaction to continue to proceed forward in a free-energy favorable fashion.

VIII. Esterifying PAA $_{P\&G}$ fragments to make PSAs

PAA $_{P\&G\_5\%\text{-}0min}$: To a 75 mL pressure vessel, 2-EHOH (3.80 mL, 24.3 mmol, 5.00 equiv.) and H_2SO_4 (0.065 mL, 1.21 mmol, 0.25 equiv.) were added and stirred at 120 °C. While stirring, PAA $_{P\&G\_5\%\text{-}0min}$ (350 mg, 4.90 mmol, 1.00 equiv.) was subsequently added and the vessel was sealed and stirred for 10 h at 120 °C. Thereafter, the vessel was cooled in a rt water bath. The poly(2-ethylhexyl acrylate)$_{P\&G\text{-}0min}$ ((P(2-EHA))$_{P\&G\_5\%\text{-}0min}$) was isolated by precipitating into MeOH (20 mL) and removing the supernatant. Then, the polymer was purified by dissolving in minimal amounts of THF (5 mL), precipitating into MeOH (20 mL), and removing the supernatant. This process was repeated three times. The resulting solid was dried under high vacuum at 80 °C for 10 h. The isolated yield was 81%. A portion of the P(2-EHA)$_{P\&G\_5\%\text{-}0min}$ (600 mg) was used for frequency sweep measurements.

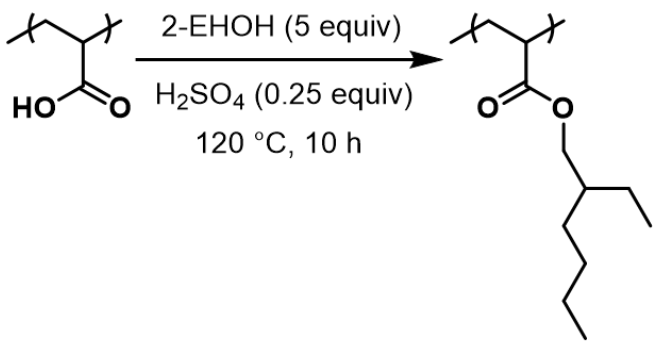

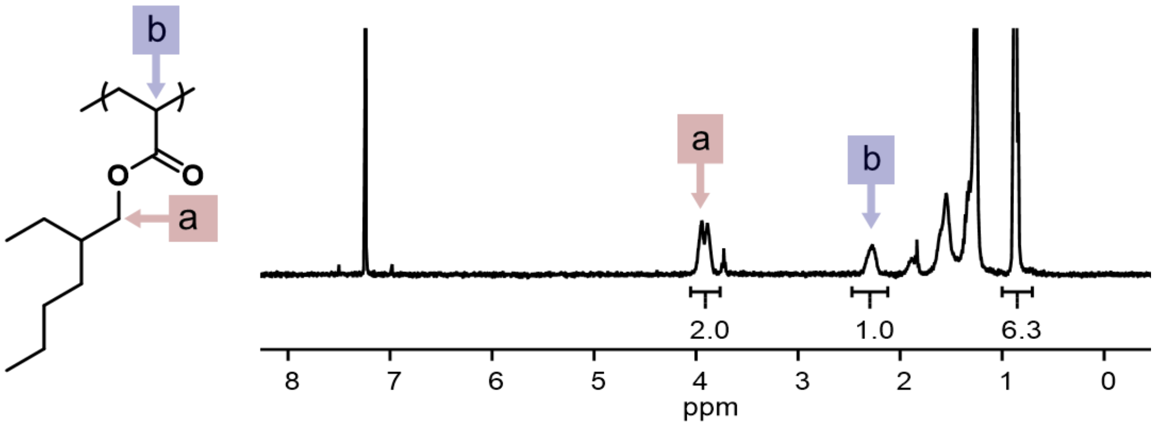

Supplementary Fig. 23. ^{1}H NMR spectrum for P(2-EHA)$_{P\&G\_5\%\text{-}0min}$ (500 MHz, $CDCl_3$).

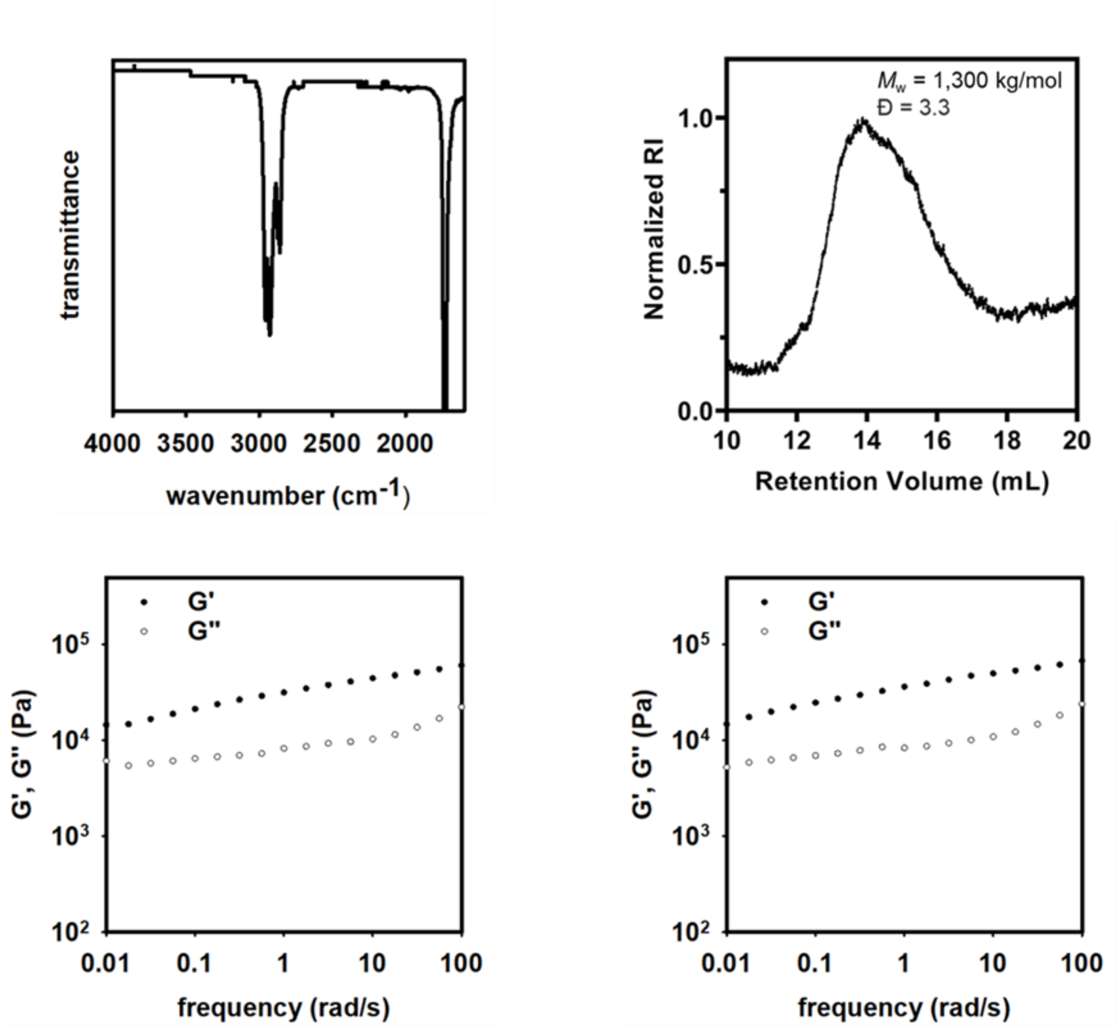

Supplementary Fig. 24. IR spectrum (top left), SEC trace (top right), and frequency sweeps (bottom) of P(2-EHA)$_{P\&G\_5\%\text{-}0min}$, made via esterifying decrosslinked PAA$_{P\&G_5\%\text{-}0min}$.

P(2-EHA)$_{P\&G\_5\%-2min}$: To a 75 mL pressure vessel, 2-EHOH (4.34 mL, 27.8 mmol, 5.00 equiv.) and H_2SO_4 (0.074 mL, 1.39 mmol, 0.25 equiv.) were added and stirred at 120 °C. While stirring, PAA $_{P\&G\_5\%-2min}$ (400 mg, 5.60 mmol, 1.00 equiv.) was subsequently added and the vessel was sealed and stirred for 10 h at 120 °C. Thereafter, the vessel was cooled in a rt water bath. The poly(2-ethylhexyl acrylate)$_{P\&G\_5\%-2min}$ ((P(2-EHA))$_{P\&G\_5\%-2min}$) was isolated by precipitating into MeOH (20 mL) and removing the supernatant. Then, the polymer was purified by dissolving in minimal amounts of THF (5 mL), precipitating into MeOH (20 mL), and removing the supernatant. This process was repeated three times. The resulting solid was dried under high vacuum at 80 °C for 10 h. The isolated yield was 78%. A portion of the P(2-EHA)$_{P\&G\_5\%-2min}$ (600 mg) was used for frequency sweep measurements.

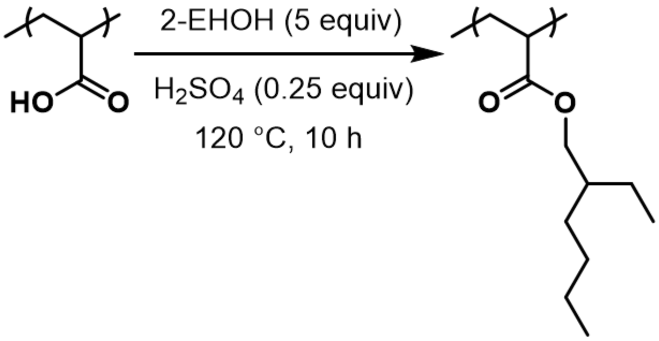

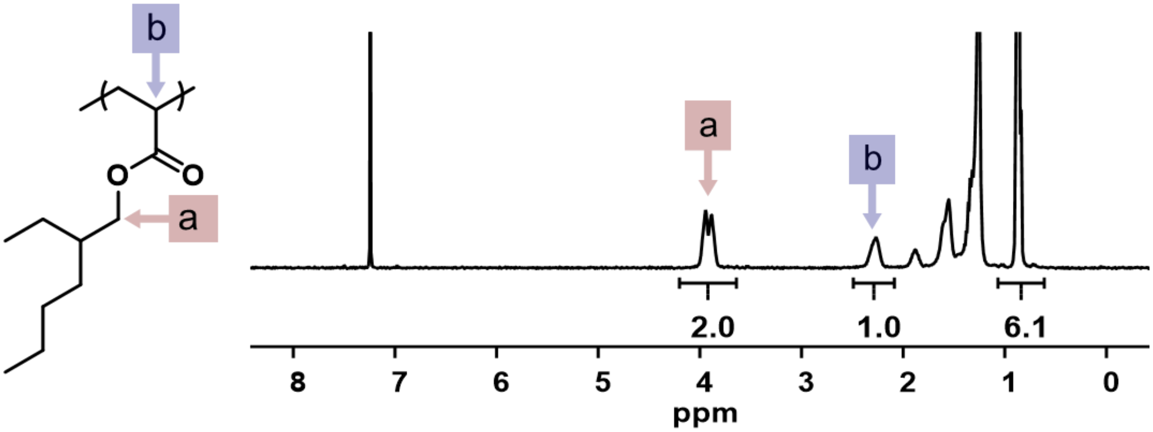

Supplementary Fig. 25. ^{1}H NMR spectrum for P(2-EHA)$_{P\&G\_5\%-2min}$ (500 MHz, $CDCl_3$).

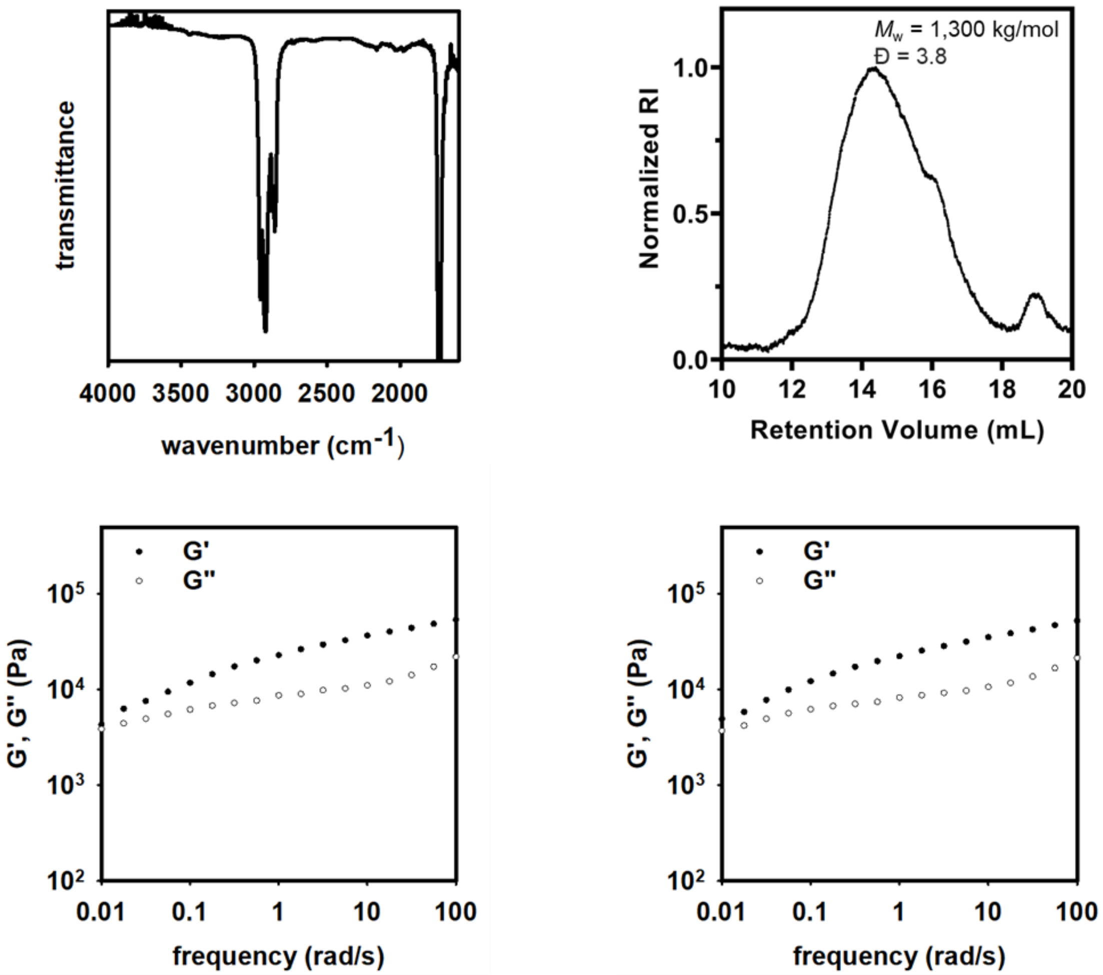

Supplementary Fig. 26. IR spectrum (top left), SEC trace (top right), and frequency sweeps (bottom) of P(2-EHA)$_{P\&G\_5\%\text{-}2min}$ made by esterifying decrosslinked PAA$_{P\&G_5\%\text{-}2min}$.

P(2-EHA)$_{P\&G\_2.5\%-1min}$: To a 75 mL pressure vessel, 2-EHOH (6.51 mL, 41.6 mmol, 5.00 equiv.) and H_2SO_4 (0.111 mL, 2.08 mmol, 0.25 equiv.) were added and stirred at 120 °C. While stirring, PAA$_{P\&G_2.5\%-1min}$ (600 mg, 10.4 mmol, 1.0 equiv.) was subsequently added and the vessel was sealed and stirred for 10 h at 120 °C. Thereafter, the vessel was cooled in a rt water bath. The poly(2-ethylhexyl acrylate)$_{P\&G\_2.5\%-1min}$ ((P(2-EHA))$_{P\&G\_2.5\%-1min}$) was isolated by precipitating into MeOH (20 mL) and removing the supernatant. Then, the polymer was purified by dissolving in minimal amounts of THF (5 mL), precipitating into MeOH (20 mL), and removing the supernatant. This process was repeated three times. The resulting solid was dried under high vacuum at 80 °C for 10 h. The isolated yield 73%. A portion of the P(2-EHA)$_{P\&G\_2.5\%-1min}$ (600 mg) was used for frequency sweep measurements.

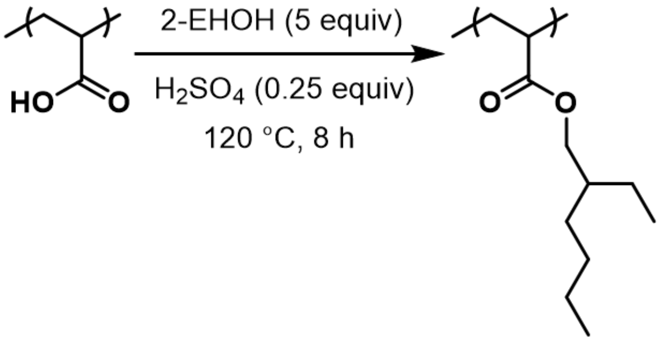

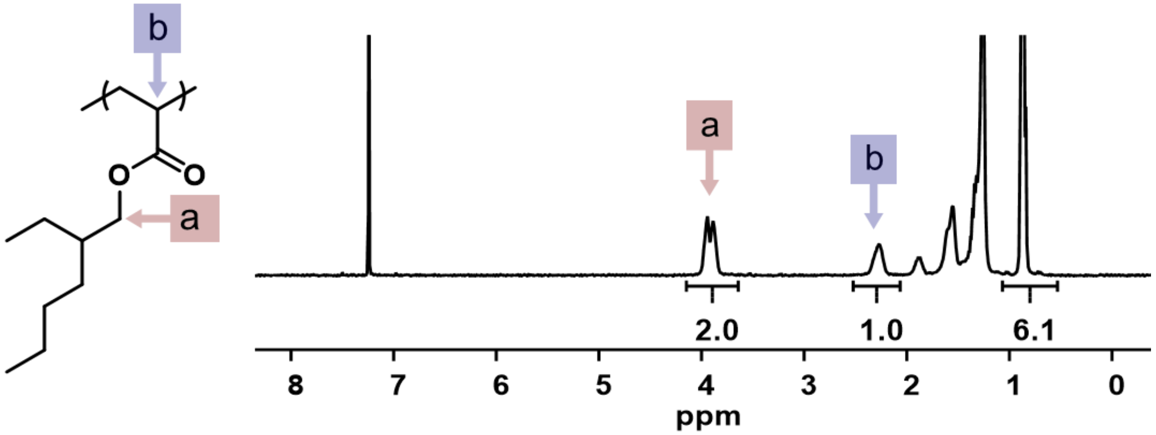

Supplementary Fig. 27. ^1H NMR spectrum for P(2-EHA)$_{P\&G\_2.5\%-1min}$ (500 MHz, $CDCl_3$).

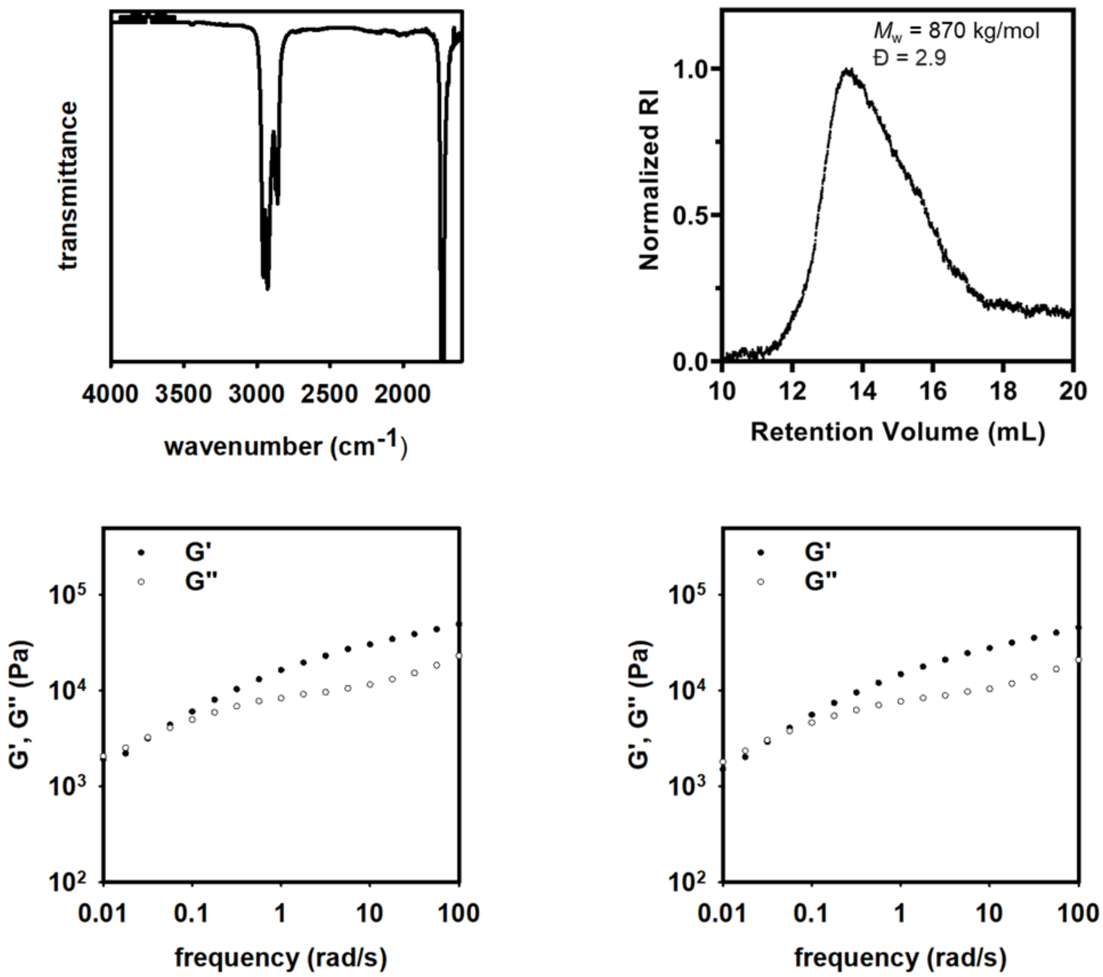

Supplementary Fig. 28. IR spectrum (top left), SEC trace (top right), and frequency sweeps (bottom) of P(2-EHA) $_{P\&G\_2.5\%\text{-}1min}$, made by esterifying decrosslinked PAA $_{P\&G\_2.5\%\text{-}1min}$.

IX. Life cycle assessment

We applied a life cycle assessment (LCA) to evaluate the environmental impacts of these novel repurposing methods in comparison to business-as-usual production of poly(2-ethylhexyl acrylate) (P(2-EHA)).

Methods

Modeling of different disposal scenarios was done using the software SimaPro version 9.0.0.48 and the ecoinvent database version 3.5[16] and impacts were calculated using the ReCiPe 2016 v1.1 midpoint method, hierarchist version.[17] Processes and material production were assumed to take place in the US and therefore all ecoinvent processes were specific to a US scenario. LCA results for all impact categories are reported, paying specific attention to cumulative energy demand (CED) and global warming potential (GWP).

Goal and scope

The main goal of the LCA was to quantify the potential environmental impacts of the novel recovery processes and compare them to commercial production of P(2-EHA). Four total scenarios were investigated: 1) the reference scenario of P(2-EHA) production from ethanol and acrylic acid, 2) hydrolysis of reused superabsorbent poly(acrylic acid) with sonication for 1 min followed by esterification 3) hydrolysis of reused superabsorbent poly(acrylic acid) with sonication for 2 min followed by esterification, and 4) hydrolysis with no sonication, followed by esterification. For scenarios 2–4, we assume that superabsorbent poly(acrylic acid) is recovered from used diapers. Given the hypothetical nature of this LCA, we did not take impacts regarding transportation or distribution of the recovered superabsorbent poly(acrylic acid) or any other materials into account. In this LCA, the functional unit is 5000 mg of P(2-EHA). The inventory data for the sonication and no-sonication processes are specific to the production of 5000 mg of P(2-EHA); therefore, we compare these scenarios to business-as-usual production of 5000 mg of P(2-EHA).

Inventory Data

The following tables contain the inputs for the LCA scenarios. Energy to model the 2-ethylhexanol to produce P(2-EHA) and the 2-ethylhexanol used in the sonication and no sonication scenarios was taken from Poulikidou et al.[18], given the absence of data in ecoinvent. In the P(2-EHA) scenario, we calculate that 391 g of acrylic acid are required in tandem with 1 kg 2-ethylhexanol (1000 grams) to produce P(2-EHA) based on the relative composition of P(2-EHA). In the sonication and no-sonication scenarios, we assume that the excess of the 5 equiv. of 2-ethylhexanol used is recoverable and therefore only included the emissions associated with the use of 1 equiv. of 2-ethylhexanol. Importantly, given the energy required in 2-ethylhexanol production, failure to re-use 2-ethylhexanol will likely favor business-as-usual production of (P-2EHA) over the repurposing scenarios. Emissions data for the extraction of superabsorbent poly(acrylic acid) from diapers was taken from a recent LCA of novel diaper recycling technology in Japan.[19] We account for the collection, separation, waste water treatment, organic acid recovery, and ozone treatment required to recover (~80%) of the superabsorbent poly(acrylic acid) in one diaper. Since LCA data for these steps was reported per diaper, we multiplied each value by 19%, the percent composition of superabsorbent poly(acrylic acid) . These numbers were then adjusted to account for 80% recovery (i.e., per 9.04 g superabsorbent poly(acrylic acid) not the 11.3 g superabsorbent poly(acrylic acid) in a diaper before recycling). Lastly, to account for the freeze-drying of superabsorbent poly(acrylic acid) in our process, we included data from a freeze-drying LCA.[20]

Supplementary Table 11. Inventory data for reference P(2-EHA scenario)

material	amount	comments
poly(2-ethylhexyl acrylate)*	5000 mg	
*modeled as follows; per 1 kg P(2-EHA)		
electricity, US grid	117 MJ	Taken from Poulikidou et al., 2019 (18)
acrylic acid	391 g	Required acrylic acid to make 1kg P(2-EHA) based on fraction of acrylic acid in P(2-EHA)

Supplementary Table 12. *Inventory data for sonication scenarios*

material	amount	comments
sulfuric acid for protonation and decrosslinking	3.91 g	
sulfuric acid for esterification	0.65 g	
2-ethylhexanol [a]	0.00346 kg	1 equivalent. Assuming remainder recycled
electricity, low voltage, US grid for sonication	34.8 kJ	For sonication 2 min scenario: 2 min, 290 watts
or --->	33.6 kJ	For sonication 1 min scenario: 1 min, 280 watts
electricity, low voltage, US grid for decrosslinking heat	20.92 kJ	
electricity, low voltage, US grid for esterification heat	2.976 kJ	
superabsorbent poly(acrylic acid) extraction [b]	2500mg	
lyophilization [c]	50g	
[a] modeled as follows; per 1 kg 2-ethylhexanol		Taken from Poulikidou et al., 2019 (18)
electricity, US grid	117 MJ	
[b] modeled as follows; per 9.04 grams of superabsorbent poly(acrylic acid) (80% recovery from 1 diaper)		Taken from Itsubo et al., 2019 (19)
land use	7.9 cm^2a	
CO_2 eq	6.8 g	
water	0.22 m^3	
[c] modeled as follows; per 2.43 kg of water		Taken from Prosapio et al., 2017 (20)
electricity, US grid	1.98E-01	
electricity, US grid	4.36E-01	
electricity, US grid	2.67E-01	
wastewater to treatment	2437 cm^3	

Supplementary Table 13. Inventory data for no sonication scenario

material	amount	comments
sulfuric acid for protonation and decrosslinking	3.91 g	
sulfuric acid for esterification	0.65 g	
2-ethylhexanol [a]	0.00346 kg	1 equivalent. Assuming remainder recycled
electricity, low voltage, US grid for decrosslinking heat	20.92 kJ	
electricity, low voltage, US grid for esterification heat	2.976 kJ	
superabsorbent poly(acrylic acid) extraction [b]	2500mg	
lyophilization [c]	50g	
[a] modeled as follows; per 1 kg 2-Ethylhexanol		Taken from Poulikidou et al., 2019 (18)
Electricity, US grid	117 MJ	
[b] modeled as follows; per 9.04 grams of superabsorbent poly(acrylic acid) (80% recovery from 1 diaper)		Taken from Itsubo et al., 2019 (19)
Land use	7.9 cm^2a	
CO2eq	6.8 g	
Water	0.22 m^3	
[c] modeled as follows; per 2.43 kg of water removed		Taken from Prosapio et al., 2017 (20)
electricity, US grid	1.98E-01	
electricity, US grid	4.36E-01	
electricity, US grid	2.67E-01	
wastewater to treatment	2437 cm^3	

Results

Supplementary Table 14. Impact assessment results for all four scenarios. Conditional formatting is applied for ease of comparison.

Impact Category	Unit	Industrial	2.5%_1min	5%_2min	5%_0min
Non renewable fossil	MJ	3.11E+02	2.48E+02	2.49E+02	2.30E+02
Non renewable nuclear	MJ	1.07E+02	9.10E+01	9.13E+01	8.43E+01
Non renewable biomass	MJ	5.93E-05	5.43E-05	5.45E-05	5.04E-05
Renewable biomass	MJ	4.38E+00	3.74E+00	3.75E+00	3.45E+00
Renewable wind solar geothe	MJ	5.11E-01	4.32E-01	4.33E-01	4.00E-01
Renewable water	MJ	9.59E+00	8.15E+00	8.17E+00	7.54E+00
CED	MJ	4.33E+02	3.51E+02	3.52E+02	3.25E+02
Global warming	kg CO2 eq	2.58E+01	2.18E+01	2.19E+01	2.03E+01
Stratospheric ozone depletion	kg CFC11 eq	7.04E-06	6.24E-06	6.26E-06	5.68E-06
Ionizing radiation	kBq Co-60 eq	2.40E-01	2.04E-01	2.05E-01	1.89E-01
Ozone formation Human health	kg NOx eq	4.82E-02	4.12E-02	4.13E-02	3.81E-02
Fine particulate matter formation	kg PM2.5 eq	4.45E-02	4.14E-02	4.15E-02	3.85E-02
Ozone formation Terrestrial ecosystems	kg NOx eq	4.88E-02	4.16E-02	4.17E-02	3.85E-02
Terrestrial acidification	kg SO2 eq	1.47E-01	1.37E-01	1.38E-01	1.28E-01
Freshwater eutrophication	kg P eq	1.47E-03	1.31E-03	1.32E-03	1.20E-03
Marine eutrophication	kg N eq	9.40E-05	1.42E-04	1.43E-04	1.36E-04
Terrestrial ecotoxicity	kg 1,4-DCB	9.36E+00	1.09E+01	1.09E+01	8.86E+00
Freshwater ecotoxicity	kg 1,4-DCB	1.52E-02	1.33E-02	1.33E-02	1.22E-02
Marine ecotoxicity	kg 1,4-DCB	2.68E-02	2.46E-02	2.47E-02	2.22E-02
Human carcinogenic toxicity	kg 1,4-DCB	7.93E-02	9.51E-02	9.59E-02	7.43E-02
Human non carcinogenic toxicity	kg 1,4-DCB	2.79E+00	2.52E+00	2.53E+00	2.29E+00
Land use	m2a crop eq	2.27E-01	1.97E-01	1.97E-01	1.82E-01
Mineral resource scarcity	kg Cu eq	1.75E-02	1.86E-02	1.87E-02	1.61E-02
Fossil resource scarcity	kg oil eq	6.78E+00	5.40E+00	5.42E+00	5.00E+00
Water consumption	m3	9.53E+01	9.38E+01	9.40E+01	8.77E+01

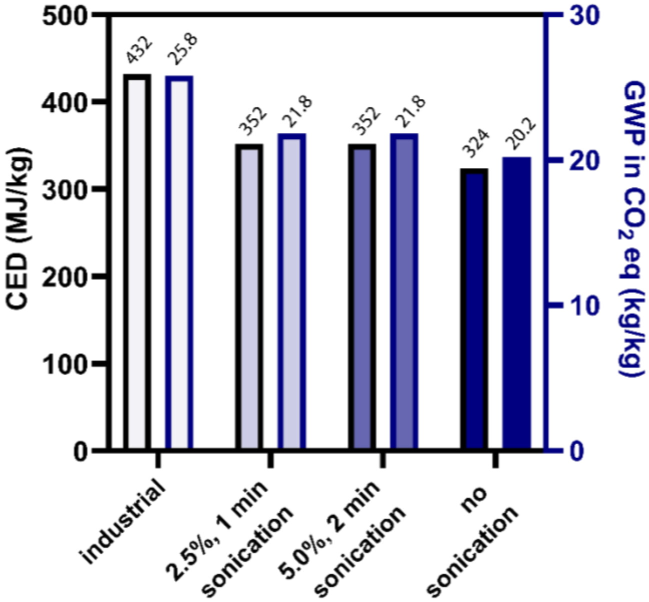

Supplementary Fig. 29. Plot of global warming potential and cumulative energy demand for the different LCA scenarios.

LCA results indicate that the PSA repurposing scenarios show improvement in almost all LCA impact categories. Moderate decreases in GWP and CED are indicated for the two sonication scenarios in comparison to conventional P(2-EHA) (~13% reduction in GWP and ~18% reduction in CED). The no sonication scenario (5%, 0min) outperforms the sonication scenarios, unsurprisingly, given the reduction in electricity required. More specifically, the no sonication scenario shows a ~20% reduction in GWP and ~25% reduction in CED compared to conventional P(2-EHA).

Supplementary References

---

(1) Moriguchi, T. & Arita, Y. Process for Producing Acrylic Acid. U.S. Patent 8404887B2. March 26, 2013.

(2) Christ, C. D., Mark, A. E. & Van Gunsteren, W. F. Basic Ingredients of Free Energy Calculations: A Review. *J. Comput. Chem.* **31**, 1569–1582 (1990). https://doi.org/10.1002/jcc.21450.

(3) Hanwell, M. D., Curtis, D. E., Lonie, D. C., Vandermeerschd, T., Zurek, E. & Hutchison, G. R. Avogadro: An Advanced Semantic Chemical Editor, Visualization, and Analysis Platform. *J. Cheminform.* **4**, 17 (2012). https://doi.org/10.1186/1758-2946-4-17.

(3) Martínez, L., Andrade, R., Birgin, E. G. & Martínez, J. M. PACKMOL: A Package for Building Initial Configurations for Molecular Dynamics Simulations. *J. Comput. Chem.* **30**, 2157–2164 (2009). https://doi.org/10.1002/jcc.21224.

(5) Jorgensen, W. L., Chandrasekhar, J., Madura, J. D., Impey, R. W. & Klein, M. L. Comparison of Simple Potential Functions for Simulating Liquid Water. *J. Chem. Phys.* **79**, 926–935 (1983). https://doi.org/10.1063/1.445869.

(6) Vanommeslaeghe, K., Hatcher, E., Acharya, C., Kundu, S., Zhong, S., Shim, J., Darian, E., Guvench, O., Lopes, P., Vorobyov, I. & Mackerell, A. D. CHARMM General Force Field: A Force Field for Drug-like Molecules Compatible with the CHARMM All-Atom Additive Biological Force Fields. *J. Comput. Chem.* **31**, NA-NA (2009). https://doi.org/10.1002/jcc.21367.

(7) Yesselman, J. D., Price, D. J., Knight, J. L. & Brooks, C. L. MATCH: An Atom-Typing Toolset for Molecular Mechanics Force Fields. *J. Comput. Chem.* **33**, 189–202 (2012). https://doi.org/10.1002/jcc.21963.

(8) Brooks, B. R., Brooks, C. L., Mackerell, A. D., Nilsson, L., Petrella, R. J., Roux, B., Won, Y., Archontis, G., Bartels, C., Boresch, S., Caflisch, A., Caves, L., Cui, Q., Dinner, A. R., Feig, M., Fischer, S., Gao, J., Hodoscek, M., Im, W., Kuczera, K., Lazaridis, T., Ma, J., Ovchinnikov, V., Paci, E., Pastor, R. W., Post, C. B., Pu, J. Z., Schaefer, M., Tidor, B., Venable, R. M., Woodcock, H. L., Wu, X., Yang, W., York, D. M. & Karplus, M. CHARMM: The Biomolecular Simulation Program. *J. Comput. Chem.* **30***,* 1545–1614 (2009). https://doi.org/10.1002/jcc.21287.

(9) Hynninen, A.-P. & Crowley, M. F. New Faster CHARMM Molecular Dynamics Engine. *J. Comput. Chem.* **35**, 406–413 (2014). https://doi.org/10.1002/jcc.23501.

(10) Darden, T., York, D. & Pedersen, L. Particle Mesh Ewald: An N·log(N) Method for Ewald Sums in Large Systems. *J. Chem. Phys.* **98**, 10089–10092 (1993). https://doi.org/10.1063/1.464397.

(11) Essmann, U., Perera, L., Berkowitz, M. L., Darden, T., Lee, H. & Pedersen, L. G. A Smooth Particle Mesh Ewald Method. *J. Chem. Phys.* **103**, 8577–8593 (1995). https://doi.org/10.1063/1.470117.

(12) Huang, Y., Chen, W., Wallace, J. A. & Shen, J. All-Atom Continuous Constant PH Molecular Dynamics with Particle Mesh Ewald and Titratable Water. *J. Chem. Theory Comput.* **12**, 5411–5421 (2016). https://doi.org/10.1021/acs.jctc.6b00552.

(13) Shirts, M. R. & Chodera, J. D. Statistically Optimal Analysis of Samples from Multiple Equilibrium States. *J. Chem. Phys.* **129**, 124105 (2008). https://doi.org/10.1063/1.2978177.

(14) Vilseck, J. Z., Sohail, N., Hayes, R. L. & Brooks, C. L. Overcoming Challenging Substituent Perturbations with Multisite λ-Dynamics: A Case Study Targeting β-Secretase 1. *J. Phys. Chem. Lett.* **10**, 4875–4880 (2019). https://doi.org/10.1021/acs.jpclett.9b02004.

(15) Hayes, R. L., Armacost, K. A., Vilseck, J. Z. & Brooks, C. L. Adaptive Landscape Flattening Accelerates Sampling of Alchemical Space in Multisite λ Dynamics. *J. Phys. Chem. B.* **121**, 3626–3635 (2017). https://doi.org/10.1021/acs.jpcb.6b09656.

(16) Wernet, G., Bauer, C., Steubing, B., Reinhard, J., Moreno-Ruiz, E. & Weidema, B. The Ecoinvent Database Version 3 (Part I): Overview and Methodology. *Int. J. Life Cycle Assess.* **21**, 1218–1230 (2016). https://doi.org/10.1007/s11367-016-1087-8.

(17) LCIA: the ReCiPe Model.

(18) Poulikidou, S., Heyne, S., Grahn, M. & Harvey, S. Lifecycle Energy and Greenhouse Gas Emissions Analysis of Biomass-Based 2-Ethylhexanol as an Alternative Transportation Fuel. *Energy Sci. Eng.* **7**, 851–867 (2019). https://doi.org/10.1002/ese3.315.

(19) Itsubo, N., Wada, M., Imai, S., Myoga, A., Makino, N. & Shobatake, K. Life Cycle Assessment of the Closed-Loop Recycling of Used Disposable Diapers. *Resources* **9**, 34 (2020). https://doi.org/10.3390/resources9030034.

(20) Prosapio, V., Norton, I. & De Marco, I. Optimization of Freeze-Drying Using a Life Cycle Assessment Approach: Strawberries' Case Study. *J. Clean. Prod.* **168**, 1171–1179 (2017). https://doi.org/10.1016/j.jclepro.2017.09.125.
